# A modular phosphate tether-mediated divergent strategy to complex polyols

**DOI:** 10.3762/bjoc.10.242

**Published:** 2014-10-07

**Authors:** Paul R Hanson, Susanthi Jayasinghe, Soma Maitra, Cornelius N Ndi, Rambabu Chegondi

**Affiliations:** 1 Department of Chemistry, University of Kansas, 1251 Wescoe Hall Drive, Lawrence, KS 66045-7582, USA

**Keywords:** phosphate-tether, one-pot, sequential processes, organophosphorus, polyol, stereodivergent

## Abstract

An efficient and divergent synthesis of polyol subunits utilizing a phosphate tether-mediated, one-pot, sequential RCM/CM/reduction process is reported. A modular, 3-component coupling strategy has been developed, in which, simple “order of addition” of a pair of olefinic-alcohol components to a pseudo-*C*_2_-symmetric phosphoryl chloride, coupled with the RCM/CM/reduction protocol, yields five polyol fragments. Each of the product polyols bears a central 1,3-*anti-*diol subunit with differential olefinic geometries at the periphery.

## Introduction

**Scheme 1:**
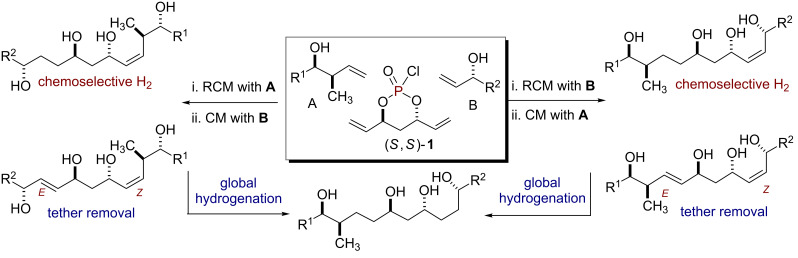
The 3-component coupling strategy.

1,3-*anti-*Diol subunits are a central component in several potent biologically active polyketides [1–4]. This prevalence has led to the development of various synthetic methods for their construction [5]. In particular, divergent strategies are ideal for analog generation [6–11], which in turn, can enhance the quality of screening decks in early phase drug discovery. One aim of divergent synthetic strategies is to produce multiple scaffolds from a single set of starting materials [12]. In this regard, one-pot, sequential processes [13–16] are well suited to address this challenge by forming multiple bonds and stereocenters, while invoking step- [17], atom- [18–21], green- [22–23], and pot economy [24–26]. We have previously reported phosphate tether-mediated strategies to streamline the synthesis of 1,3-*anti*-diol containing natural products, including recent reports employing one-pot, sequential protocols [27–32]. Herein, we report a modular, divergent approach to construct advanced polyol intermediates **10**–**14** and **17**–**21** in one- or two-pot sequences utilizing the innate properties of a phosphate tether. Taken collectively, this modular, divergent 3-component coupling strategy generates five polyol fragments, bearing differential *Z*- and *E*-olefins, from a pair of olefinic-alcohol components **A** and **B** and a pseudo-*C*_2_-symmetric phosphoryl chloride (*S*,*S*)-**1**. Moreover, the method relies on simple "order of addition" of components for the phosphoryl coupling, ring-closing metathesis (RCM) and cross metathesis (CM) steps of the process as outlined in Scheme 1. This protocol further highlights the utility of phosphate tether mediated desymmetrization of *C*_2_-symmetric 1,3-*anti-*diene-diol subunit to generate polyol scaffolds which would otherwise be difficult to produce via (*Z)-*and (*E)-*selective CM of 1,3-*anti*-diene-diol subunits with olefinic-alcohol components.

## Results and Discussion

The titled divergent strategy was initiated during efforts to further explore the utility of phosphate tethers. Previous reports emphasized the utilization of phosphate tethers in chemo- and diastereoselective reactions including one-pot, sequential RCM/CM/H_2_ protocols and their applications in total synthesis of various natural products [27–32]. In addition, the scope of phosphate-tethered methods was further expanded via extensive RCM studies of different triene subunits utilizing stereochemically divergent olefin partners [33]. Recently, the potential of phosphate tethered facilitated processes were highlighted in the pot-economical and efficient total synthesis of the antifungal agent strictifolione, whereby two consecutive phosphate tether-mediated, one-pot, sequential protocols were employed [34].

**Figure 1:**
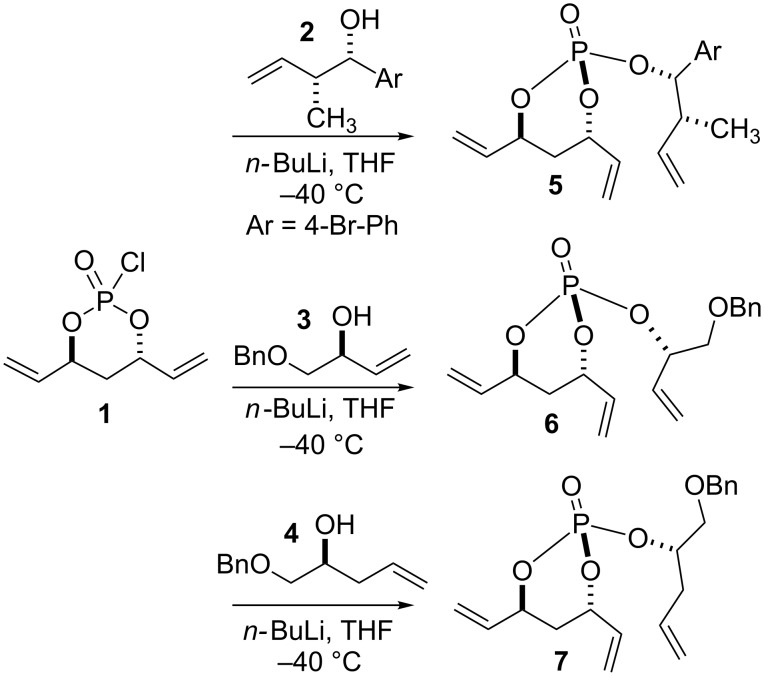
Synthesis of trienes **5**–**7**.

The requisite trienes **5**–**7** for this study were generated via our previously reported coupling of the pseudo-*C*_2_-symmetric phosphoryl chloride (*S,S*)-**1** with the olefinic alcohol components **2**–**4** [27–32]. The alcohol substrates are carefully chosen to incorporate exo-allylic methyl groups since previous RCM studies [33] showed that the productive RCM for 8-membered ring formation was observed only for the *S*,*S*- configured trienes in the presence of an exo-methyl group at the allylic position (Figure 1).

**Scheme 2:**
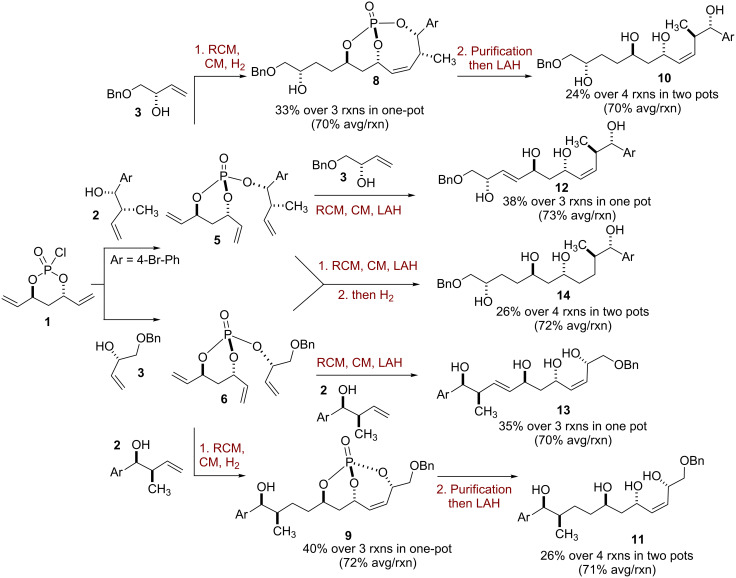
Synthesis of polyols **10**–**14** in one-, two-pot sequential protocols.

Initial attempts were focused on generating the first set of five polyols starting from trienes **5** and **6** in a two-pot operation (Scheme 2). The first operation entailed a one-pot, sequential RCM/CM/chemoselective hydrogenation protocol [32], yielding two bicyclo[*n*.3.1]phosphate intermediates **8** and **9**, and a second pot LiAlH_4_ reduction to provide the *Z*-configured tetraol subunits **10** and **11**. Trienes **5** and **6** were generated via coupling with alcohol partners **2** and **3**, respectively, and the divergent aspect of the method was introduced by simple switching of the olefinic partners in the subsequent CM reaction to afford five differentiated polyols starting from three coupling partners.

In this regard, triene **5** was first subjected to RCM in the presence of the Hoveyda–Grubbs II (**HG**-**II**) catalyst [35–37] in refluxing CH_2_Cl_2_, followed by solvent concentration and CM with allylic alcohol **3** in refluxing CH_2_Cl_2_ for two hours. It was observed that the use of CH_2_Cl_2_ was critical for the successful CM reactions in order to avoid the formation of isomerized ketone byproducts. Subsequent chemoselective diimide reduction with *o*-nitrobenzenesulfonylhydrazine (*o*-NBSH) [38–40] in CH_2_Cl_2_ at room temperature afforded bicyclo[5.3.1]phosphate **8** in 33% overall yield, representing а 70% average yield/reaction in the one-pot, sequential protocol (Scheme 2). Subsequent treatment of **8** with LiAlH_4_ furnished the tetraol **10** in 24% overall yield over the course of four reaction steps carried out in two pots, representing a 70% average yield per reaction.

Similarly, starting with the triene **6**, a one-pot RCM/CM/chemoselective H_2_ was performed to obtain the bicyclo[4.3.1]phosphate **9** in 40% yield over 3 reaction steps in a one-pot operation (72% avg/rxn). In this example, the RCM reaction was performed in dicholoroethane (DCE) at 70 °C for 2 h, since lower reactivity was observed in CH_2_Cl_2_. Subsequently, phosphate **9** was treated with LiAlH_4_ to generate tetraol **11** in 26% overall yield in the four reactions carried out in two pots, representing a 71% average yield per reaction.

Next, a one-pot RCM/CM/LAH protocol was established to obtain two additional tetraol subunits possessing both *E-* and *Z-*olefin geometries. Triene **5**, was subjected to an RCM reaction, followed by a CM reaction with allylic alcohol **3**. After removing the solvent, the CM product was treated with LiAlH_4_ to produce tetraol **12** in 38% yield over three reaction steps in the one-pot, sequential process (73% avg/rxn) (Scheme 2). Similarly, triene **6** was subjected to an RCM reaction, followed by CM with homoallylic alcohol **2**, and subsequent treatment with LiAlH_4_ to afford tetraol **13** in 35% yield over the three reaction steps, representing a 70% avg/rxn in the one-pot, sequential method.

This RCM/CM/LAH procedure was further merged with global hydrogenation, whereby the resulting tetraols **12** and **13**, after one-pot, sequential RCM/CM/LAH protocol, were separately treated with *o*-NBSH to obtain tetraol **14** in 26% yield over the four reaction steps in a two-pot operation starting from triene **5** (72% avg/rxn). Utilizing this two-pot sequential protocol, the same tetraol **14** was obtained starting from two different trienes (**5** and **6**) and reacting with two different CM partners. It should be noted, that after phosphate tether removal, treatment of tetraol with *o*-NBSH (20 equiv), resulted in the global reduction of both *E-* and *Z*-olefins in very good yields, while in contrast, diimide reduction in the presence of phosphate intermediates did not hydrogenate the endocyclic olefin even when large excesses of diimide reagent were employed (30 equiv). This empirical result further substantiates the protecting group ability of the phosphate tether for the endocyclic *Z-*olefin.

**Scheme 3:**
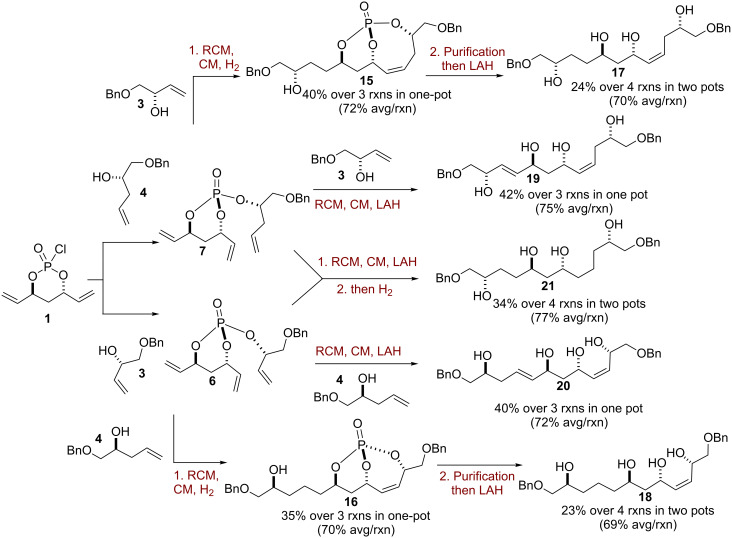
Synthesis of polyols **17**–**21** in one-, two-pot sequential protocols.

Next, our attempts were focused on generating the second set of five polyols starting from trienes **6** and **7**. Utilizing the aforementioned strategy detailed in Scheme 2, triene **7** was subjected to the one-pot, sequential RCM/CM/chemoselective H_2_ procedure and subsequent LiAlH_4_ reduction to generate tetraol **17** in 24% yield over 4 reaction steps in a two-pot operation (70% avg/rxn) (Scheme 3). Further, starting with the same triene **6**, which was previously used in Scheme 2, but utilizing a different cross-metathesis partner (homoallylic alcohol **4**), a different tetraol **18** was generated in 23% yield over the four reaction steps in a two-pot operation (69% avg/rxn) (Scheme 3).

In a similar manner, starting with triene **7**, RCM and subsequent CM with allylic alcohol **3**, followed by tether removal with LiAlH_4_, were performed to obtain tetraol **19** in 42% yield over three reaction steps in the one-pot, sequential operation (75% avg/rxn). Triene **6** was next subjected to RCM, followed by CM with homoallylic alcohol **4** and LiAlH_4_ reduction to furnish tetraol **20** in an overall 40% yield over three reaction steps in a one-pot operation (72% avg/rxn). Tetraols **19** and **20** were separately subjected to a global hydrogenation using *o*-NBSH to afford tetraol **21** in 34% yield over four reaction steps in a two-pot operation starting from triene **6** (77% avg/rxn).

## Conclusion

In conclusion, we have reported one- or two-pot sequential methods mediated by a phosphate tether to generate a diverse array of polyol molecules utilizing readily prepared trienes **5**, **6** and **7** and CM partners **2**, **3**, and **4**. This divergent method was established by taking advantage of the innate properties of phosphate tethers to provide efficient syntheses of the stereochemically-rich polyol subunits **10–14** and **17–21**.

## Supporting Information

File 1 Experimental section.
