# Supplementary material for: A modular phosphate tether-mediated divergent strategy to complex polyols
Source: Beilstein J Org Chem. 2014 Oct 7;10:2332–7. doi: 10.3762/bjoc.10.242 (PMC4187035; doi:10.3762/bjoc.10.242)

Supporting Information

for

A modular phosphate tether-mediated divergent strategy to complex polyols

Paul R. Hanson*, Susanthi Jayasinghe‡, Soma Maitra‡, Cornelius N. Ndi and Rambabu Chegondi

Address: Department of Chemistry, University of Kansas, 1251 Wescoe Hall Drive, Lawrence, KS 66045-7582, USA

Email: Paul R. Hanson - phanson@ku.edu

*Corresponding author

‡Equal contributors

Experimental section

General Methods: S1 to S5
Experimental Data: S6 to S14
^{1}H NMR, ^{13}C NMR, ^{31}P NMR spectra: S15 to S32

All reactions were carried out in an oven- or flame-dried glassware under argon atmosphere using standard gas-tight syringes, cannulae, and septa. Stirring was achieved with oven-dried magnetic stir bars. Et_2O, THF and CH_2Cl_2 were purified by passage through a purification system (Solv-Tek) employing activated Al_2O_3 (Pangborn, A. B.; Giardello, M. A.; Grubbs, R. H.; Rosen, R. K.; Timmers, F. J. Safe and Convenient Procedure for Solvent Purification *Organometallics*, **1996**, *15*, 1518–1520). Et_3N was purified by passage over basic alumina and stored over KOH. Butyllithium was purchased from Aldrich and titrated prior to use. All olefin metathesis catalysts were acquired from Materia and used without further purification. Flash column chromatography was performed with Sorbent Technologies (30930M-25, Silica Gel 60A, 40-63 μm) and thin layer chromatography was performed on silica gel $60F_{254}$ plates (EM-5717, Merck). Deuterated solvents were purchased from Cambridge Isotope laboratories. ^{1}H and ^{13}C NMR spectra were recorded in $CDCl_3$ (unless otherwise mentioned) on a Bruker DRX-500 spectrometer operating at 500 MHz, and 125 MHz, respectively and calibrated to the solvent peak. ^{31}P NMR spectra was recorded on Bruker DRX-400 spectrometer operating at 162 MHz. High-resolution mass spectrometry (HRMS) was recorded on a LCT Premier Spectrometer (Micromass UK Limited) operating on ESI (MeOH). Observed rotations at 589 nm, were measured using AUTOPOL IV Model automatic polarimeter. IR was recorded on Shimadzu FTIR-8400S instrument.

General procedure for RCM/CM/hydrogenation (Procedure A)

To a stirring solution of triene (*S*,*S*) in freshly distilled, freeze-degas-thawed dichloroethane [1] (0.007 M) was added Hoveyda-Grubbs 2nd Gen. catalyst (HG-II) (3 mol %) and the reaction was refluxed for 2 hours. After completion of RCM (monitored by TLC), the solvent was removed under reduced pressure and olefin cross partner [3–5 equivalent with respect to the triene (*S*,*S*)] dissolved in freeze-degas-thawed CH_2Cl_2 (0.1 M) was introduced , followed by addition of HG-II (3–5 mol %). It should be noted that the use of dichloromethane was critical for successful cross-metathesis reaction in order to avoid the formation of isomerized ketone byproducts. Cross-metathesis (CM) reaction in dichloroethane provided the isomerized ketone byproduct (confirmed by ^1H and ^{13}C spectra) and the cross-metathesis product with 1:1 ratio both at 70 ºC and 90 ºC. The reaction was refluxed for an additional 2–3 hours upon which the reaction showed the CM product formation along with some amounts of RCM starting material (*S*,*S*,S_P). The reaction mixture was cooled to RT and *o*-nitrobenzenesulfonylhydrazine (*o*-NBSH) (12 equiv.) and Et_3N (2 mL/g of *o*-NBSH) were added, upon which the reaction was stirred at RT overnight. The reaction mixture was quenched with sat. $NaHCO_3$ (1 mL), and diluted with CH_2Cl_2 (10 mL). The aqueous layer was washed with CH_2Cl_2 (3x5 mL) and the combined organic layers were dried (Na_2SO_4), concentrated under reduced pressure and purified using flash column chromatography.

General procedure for RCM/CM/hydrogenation (Procedure B)

To a stirring solution of triene (*S*,*S*) in freshly distilled, freeze-degas-thawed CH_2Cl_2 (0.007 M) was added HG-II (3 mol %) and the reaction was refluxed for 2 hours. After completion of RCM (monitored by TLC), the solvent was removed and olefin cross partner [3 equivalent with respect to the triene (*S*,*S*)] dissolved in freeze-degas-thawed CH_2Cl_2 (0.1 M) was introduced, followed by addition of HG-II (3 mol %). It should be noted that the use of dichloromethane was critical for successful CM reaction in order to avoid the formation of isomerized ketone byproducts. Cross-metathesis reaction in dichloroethane provided the isomerized ketone byproduct (confirmed by ^1H and ^{13}C spectra) and the CM product with 1:1 ratio both at 70 ºC and 90 ºC. The reaction was refluxed for an additional 2–3 hours upon which the reaction showed the CM product formation along with some amounts of RCM starting material (*S*,*S*,S_P). The reaction mixture was cooled to RT and *o*-nitrobenzenesulfonyl hydrazine (*o*-NBSH) (12 equiv.) and Et_3N (2 mL/g of *o*-NBSH) were added, upon which the reaction was stirred at RT overnight. The

[1] In CH_2Cl_2, the RCM appeared to be slower in the presence of HG-II.

reaction mixture was quenched with sat. $NaHCO_3$ (1 mL), and diluted with CH_2Cl_2 (10 mL). The aqueous layer was washed with CH_2Cl_2 (3x5 mL) and the combined organic layers were dried (Na_2SO_4), concentrated under reduced pressure and purified using flash column chromatography.

General procedure for RCM/CM/hydrogenation and subsequent reduction with $LiAlH_4$ (Procedure C)

The above-mentioned procedure for one-pot RCM/CM/hydrogenation was followed and the crude product was purified using flash column chromatography [2]. To a stirring solution of the hydrogenated product in dry THF (0.5 M), $LiAlH_4$ (2–4 equiv.) was added portion wise at 0 ºC and the reaction was stirred at 0 ºC for 2 hours. After the completion of reduction, the reaction was quenched via slow sequential addition of H_2O (1 mL/g of $LiAlH_4$), 10% NaOH (1 mL/g of $LiAlH_4$), and H_2O (3 mL/g of $LiAlH_4$) [Feiser workup] [3], and the ice bath was removed and the reaction was stirred for 2 h. The reaction was filtered through Celite® and washed with EtOAc (2x5 mL). The combined organic layers were concentrated under reduced pressure and purified using flash column chromatography.

General procedure for RCM/CM/$LiAlH_4$ reduction (Procedure D)

To a stirring solution of triene (*S*,*S*) in freshly distilled, freeze-degas-thawed dichloroethane (0.007 M) was added HG-II (3 mol %) and the reaction was refluxed for 2 hours. After completion of RCM (monitored by TLC), the solvent was removed and olefin cross partner [3–5 equivalent with respect to the triene (*S*,*S*)] dissolved in freeze-degas-thawed CH_2Cl_2 (0.1 M) was introduced, followed by addition of HG-II (3–6 mol %). The reaction was refluxed for an additional 2–3 hours upon which the reaction showed the CM product formation along with some amounts of RCM starting material (S,S,S_P). After the completion of CM, the solvent was evaporated under reduced pressure. The crude reaction mixture was then dissolved in dry THF (0.5 M) and cooled to 0 ºC. To this solution $LiAlH_4$ (4 equiv.) was added portion wise and the reaction was stirred at 0 ºC for 2 hours. After the completion of reduction, the reaction was quenched via slow sequential addition of H_2O (1 mL/g of $LiAlH_4$), 10% NaOH (1 mL/g of $LiAlH_4$), and H_2O (3 mL/g of $LiAlH_4$) [Feiser workup], and the ice bath was removed and the reaction was

[2] Purification was necessary at this stage for subsequent successful LAH reduction.
[3] Fieser, L.F.; Fieser, M. Reagents for Organic Synthesis Vol. 1, Wiley, New York 1967, pp 581–595.

stirred for 2 h. The reaction was filtered through Celite® and washed with EtOAc (2x5 mL). The combined organic layers were concentrated under reduced pressure and purified using flash column chromatography.

General procedure for RCM/CM/$LiAlH_4$ reduction (Procedure E)

To a stirring solution of triene (*S*,*S*) in freshly distilled, freeze-degas-thawed CH_2Cl_2 (0.007 M) was added HG-II (3 mol %) and the reaction was refluxed for 2 hours. After completion of RCM (monitored by TLC), the solvent was removed and olefin cross partner [3 equivalent with respect to the triene (*S*,*S*)] dissolved in freeze-degas-thawed CH_2Cl_2 (0.1 M) was introduced, followed by addition of Hoveyda-Grubbs 2nd Gen. catalyst (3 mol %). The reaction was refluxed for an additional 2–3 hours upon which the reaction showed the CM product formation along with some amounts of RCM starting material (S,S,S_P). After the completion of CM, the solvent was evaporated under reduced pressure. The crude reaction mixture was then dissolved in dry THF (0.5 M) and cooled to 0 ºC. To this solution $LiAlH_4$ (4 equiv.) was added portion wise and the reaction was stirred at 0 ºC for 2 hours. After the completion of reduction, the reaction was quenched via slow sequential addition of H_2O (1 mL/g of $LiAlH_4$), 10% NaOH (1 mL/g of $LiAlH_4$), and H_2O (3 mL/g of $LiAlH_4$) [Feiser workup], and the ice bath was removed and the reaction was stirred for 2 h. The reaction was filtered through Celite® and washed with EtOAc (2x5 mL). The combined organic layers were concentrated under reduced pressure and purified using flash column chromatography.

General procedure for RCM/CM/ $LiAlH_4$ reduction and subsequent global hydrogenation (Procedure F)

The above-mentioned procedure (D or E) was followed to obtain the reduced product and the crude product was dissolved in CH_2Cl_2 (0.1 M) and *o*-nitrobenzenesulfonyl hydrazine (*o*-NBSH) (20 equiv.) and Et_3N (2 mL/g of *o*-NBSH) were added, upon which the reaction was stirred at RT overnight. The reaction mixture was quenched with sat. $NaHCO_3$ (1 mL) and diluted with CH_2Cl_2 (10 mL). The aqueous layer was washed with CH_2Cl_2 (3x5 mL) and the combined organic layers were dried (Na_2SO_4), concentrated under reduced pressure and purified using flash column chromatography. At this stage, purification was performed twice in order to remove all the byproducts arising from the use of *o*-NBSH.

(4*S*,6*S*)-2-(((1*S*,2*R*)-1-(4-bromophenyl)-2-methylbut-3-en-1-yl)oxy)-4,6-divinyl-1,3,2-dioxaphosphinane 2-oxide (5)

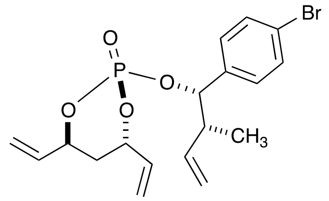

Synthesized by following the literature precedence [4].
Yield : 75%

FTIR (neat): 2962, 2927, 2349,1724, 1593, 1488, 1283, 1120, 1070, 997 cm^{-1};
Optical Rotation: $[\alpha]_D$ = + 26.7 (c = 0.075, $CHCl_3$);

^{1}**H NMR** (500 MHz, $CDCl_3$) δ 7.45 (d, J = 8.4 Hz, 2H, aromatic), 7.17 (d, J = 8.4 Hz, 2H, aromatic), 6.00 (dddd, J = 17.1, 10.6, 5.5, 0.9 Hz, 1H, H_2C=C<u>H</u>CH(OP)CH_2), 5.68 (dddd, J = 17.3, 10.6, 5.4, 1.6 Hz, 1H, H_2C=C<u>H</u>CH(OP)CH_2), 5.58 (ddd, J = 17.1, 10.5, 7.4 Hz, 1H, H_2C=C<u>H</u>CH(CH_3)CH(OP)Ar), 5.39–5.30 (m, 2H, <u>H_2</u>C=CHCH(OP)CH_2), 5.18–5.12 (m, 2H, <u>H_2</u>C=CHCH(OP)CH_2), 5.10 (d, J = 1.3 Hz, 1H, H_2C=CHCH(CH_3)C<u>H</u>(OP)Ar), 5.05 (dddd, J = 14.3, 6.8, 3.3, 1.4 Hz, 1H, H_2C=CHC<u>H</u>(OP)CH_2), 5.01–4.93 (m, 2H, <u>H_2</u>C=CHCH(CH_3)CH(OP)Ar), 4.52–4.45 (m, 1H, H_2C=CHC<u>H</u>(OP)CH_2), 2.78–2.65 (m, 1H, H_2C=CHC<u>H</u>(CH_3)CH(OP)Ar), 2.09 (dddd, J = 14.7, 8.3, 5.0, 1.5 Hz, 1H, H_2C=CHCH(OP)C<u>H_2</u>CH(OP)CH), 1.93 (dddd, J = 14.8, 5.2, 3.5, 1.9 Hz, 1H, H_2C=CHCH(OP)C<u>H_2</u>CH(OP)CH), 1.13 (d, J = 6.8 Hz, 3H, H_2C=CHCH(C<u>H_3</u>)CH(OP)Ar).

^{13}**C NMR** (126 MHz, $CDCl_3$) δ 137.9, 137.8 (d, J = 2.3 Hz), 135.2 (d, J = 3.0 Hz), 134.7 (d, J = 7.9 Hz), 131.3 (2C), 129.0 (2C), 122.2, 117.8, 117.6, 116.7, 83.5 (d, J = 6.2 Hz), 78.0 (d, J = 6.8 Hz), 75.7 (d, J = 6.0 Hz), 44.1 (d, J = 6.8 Hz), 35.0 (d, J = 7.4 Hz), 15.8.

^{31}**P NMR** (162 MHz, $CDCl_3$) δ -6.85;

HRMS: cald. for $C_{18}H_{22}BrO_4PNa$ $(M+Na)^+$ 435.0337; found 435.0325 (TOF MS ES+).

(1*S*,3*S*,4*R*,7*S*,9*R*,*Z*)-9-((*S*)-4-(benzyloxy)-3-hydroxybutyl)-3-(4-bromophenyl)-4-methyl-2,10,11-trioxa-1-phosphabicyclo[5.3.1]undec-5-ene 1-oxide (8)

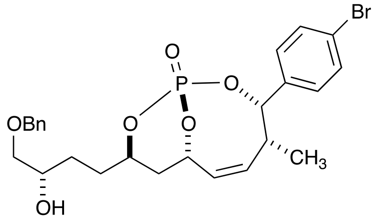

Synthesized by following procedure B

Yield: 33% over 3 reactions (70% avg/rxn)

FTIR (neat): 3411, 2962, 2927, 2873,1593, 1454, 1488, 1284, 1105, 1007, 979 cm^{-1};

Optical Rotation: $[\alpha]_D$ = - 18.3 (c = 0.59, $CHCl_3$);

^{1}**H NMR** (500 MHz, $CDCl_3$) δ 7.39 (d, J = 13.6 Hz, 2H aromatic), 7.34–7.11 (m, 5H, aromatic), 7.03 (d, J = 13.6 Hz, 2H, aromatic), 5.47 (dt, J = 11.8, 1.8 Hz, 1H, CHOPC<u>H</u>=CHCHCH$_3$CHOPAr), 5.26–5.16 (m, 3H,

[4] Chegondi, R.; Maitra, S.; Markley, J. L. Hanson, P. R. *Chem. Eur. J.* **2013**, *19*, 8088–8093.

$CH_2CHOPCH=CHCHCH_3CHOPAr$), 4.70 (t, J = 11.5 Hz, 1H, $CH_2CHOPCH_2CHOP$), 4.46 (s, 2H, CH_2OCH_2Ph), 4.01–3.92 (m, 1H, $CH=CHCHCH_3CHOPAr$), 3.77–3.68 (m, 1H, $CH_2CHOHCH_2OCH_2Ph$), 3.41 (dd, J = 9.4, 3.2 Hz, 1H $CHOHCH_2OCH_2Ph$), 3.24 (dd, J = 9.5, 7.9 Hz, 1H, $CHOHCH_2OCH_2Ph$), 2.34 (s, 1H, OH), 2.15 (ddd, J = 14.4, 11.8, 6.1 Hz, 1H, $CH_2CHOPCH_2CHOP$), 1.89–1.76 (m, 2H, $CHOHCH_2CH_2CHOPCH_2CHOP$), 1.74–1.60 (m, 2H, $CHOHCH_2CH_2CHOPCH_2CHOP$), 1.42–1.34 (m, 1H, $CHOHCH_2CH_2CHOPCH_2CHOP$), 0.69 (d, J = 6.9 Hz, 3H, $CH=CHCHCH_3CHOHAr$);

^{13}C NMR (126 MHz, $CDCl_3$) δ 137.7, 135.6 (d, J_{CP} = 13.6 Hz), 132.0, 131.1 (2C), 130.4, 129.0 (2C), 128.5 (2C), 127.9, 127.8 (2C), 122.4, 79.1 (d, J_{CP} = 4.0 Hz), 78.7 (d, J = 6.9 Hz), 78.5 (d, J_{CP} = 7.4 Hz), 74.5, 73.4, 70.3, 36.4 (d, J_{CP} = 6.5 Hz), 34.2, 32.6 (d, J_{CP} = 9.3 Hz), 28.5, 16.9;

^{31}P NMR (162 MHz, $CDCl_3$) δ -8.89;

HRMS: cald. for $C_{25}H_{30}BrO_6PNa$ $(M+Na)^+$ 559.0861; found 559.0865 (TOF MS ES+).

(1*S*,3*S*,6*S*,8*R*)-3-((benzyloxy)methyl)-8-((3*R*,4*S*)-4-(4-bromophenyl)-4-hydroxy-3-methylbutyl)-2,9,10-trioxa-1-phosphabicyclo[4.3.1]dec-4-ene 1-oxide (9):

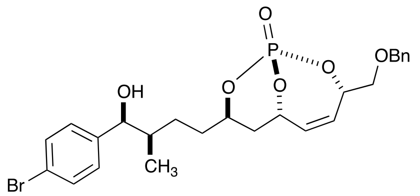

Synthesized by following procedure A

Yield: 40% over 3 reactions (72% avg/rxn)

FTIR (neat): 3400, 2974, 2285, 1630, 1288, 1209, 1101, 977, 848 cm^{-1};

Optical Rotation: $[\alpha]_D$ = + 17.34(c = 1, $CHCl_3$);

^{1}H NMR (500 MHz, $CDCl_3$) δ 7.50–7.43 (m, 2H, aromatic), 7.40–7.29 (m, 5H, aromatic), 7.21–7.16 (m, 2H, aromatic), 6.01 (ddd, J = 11.9, 3.0, 2.1 Hz, 1H, $CH=CHCHOPCH_2OBn$), 5.56 (ddd, J = 11.9, 3.9, 2.4 Hz, 1H, $CH=CHCHOPCH_2OBn$), 5.27 (dddd, J = 5.3, 5.3, 2.6, 2.6 Hz, 1H, $CH=CHCHOPCH_2OBn$), 5.18 (dddd, $J_{PH,}$ = 24.6, J_{HH} =6.2, 4.1, 1.9 Hz, 1H, $CH_2CHOPCH=CH$), 4.65–4.58 (m, 2H, CH_2OCH_2Ph), 4.58–4.47 (m, 2H, 4-BrC_6H_4CHOH, CH_2CH_2CHOP), 3.71 (ddd, J = 10.3, 5.1, 1.2 Hz, 1H, CH_2OBn), 3.61 (dd, J = 10.2, 6.0 Hz, 1H, CH_2OBn), 2.16 (ddd, J = 14.7, 12.0, 6.2 Hz, 1H, $CHOPCH_2CHOP$), 1.88 (d, J = 3.5 Hz, 1H, OH), 1.87–1.71 (m, 2H, 4-$BrC_6H_4CHOHCH(CH_3)CH_2$, $CH_2CHOPCH_2CHOP$), 1.67 (ddd, J = 14.6, 3.5, 2.0 Hz, 1H, $CHOPCH_2CHOP$), 1.58–1.48 (m, 1H, $CH_2CHOPCH_2CHOP$), 1.48–1.40 (m, 1H, $CH_2CH_2CHOPCH_2CHOP$), 1.39–1.30 (m, 1H, $CH_2CH_2CHOPCH_2CHOP$), 0.88 (d, J = 6.7 Hz, 3H, 4-$BrC_6H_4CHOHCH(CH_3)CH_2$)

^{13}C NMR (126 MHz, $CDCl_3$) δ 142.4, 137.5, 131.3 (2C), 129.9, 129.6, 128.5 (2C), 128.0 (2C), 127.9, 127.7 (2C), 121.1, 77.3 (d, J_{CP} = 6.8 Hz), 77.0, 76.8 (d, J_{CP} = 7.2 Hz), 73.5, 72.2 (d, J_{CP} = 6.1 Hz), 71.2 (d, J_{CP} = 12.2 Hz), 39.9, 34.8 (d, J_{CP} = 6.0 Hz), 33.5 (d, J_{CP} = 9.3 Hz), 27.9, 14.0;

^{31}P NMR (162 MHz, $CDCl_3$) δ -5.30;

HRMS: cald. for $C_{25}H_{30}BrO_6P$ $(M+Na)^+$ 559.0861; found (TOF MS ES+) 559.0856.

(1*S*,2*R*,5*S*,7*R*,10*S*,*Z*)-11-(benzyloxy)-1-(4-bromophenyl)-2-methylundec-3-ene-1,5,7,10-tetraol (10)

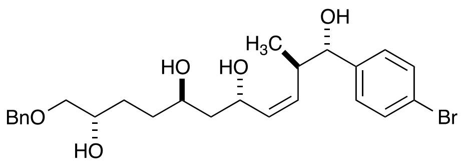

Synthesized by following procedure C

Yield: 24% over 4 reactions (70% avg/rxn)

FTIR (neat): 3367, 3335, 3061, 2921, 2852, 1595, 1519, 1456, 1093, 1072, 1026, 910 cm^{-1};

Optical Rotation: $[\alpha]_D$ =-32.3 (c = 0.33, $CHCl_3$);

^{1}H NMR (500 MHz, $CDCl_3$) δ 7.46 (d, J = 7.8 Hz, 2H, aromatic), 7.38–7.28 (m, 5H, aromatic), 7.16 (d, J = 7.8 Hz, 2H, aromatic), 5.64 (dd, J =8.5, 10.4 Hz, 1H, $CHOHC\underline{H}{=}CHCHCH_3CHOHAr$), 5.03 (dd, J =10.5, 10.6 Hz, 1H, $CHOHCH{=}C\underline{H}CHCH_3CHOHAr$), 4.70 (dd, J = 7.3, 10.7 Hz, 1H, $C\underline{H}OHCH{=}CHCHCH_3CHOHAr$), 4.60 (d, J = 4.6 Hz, 1H, $CHOHCH{=}CHCHCH_3C\underline{H}OHAr$), 4.50 (s, 2H, $CH_2OC\underline{H}_2Ph$), 4.06–3.93 (m, 1H, OH), 3.87 (m, 1H, $CH_2C\underline{H}OHCH_2CHOH$), 3.71 (dd, J = 5.7, 5.7 Hz, 1H, $CH_2C\underline{H}OHCH_2OCH_2Ph$), 3.51 (dd, J = 3.2, 9.4 Hz, 1H, $CHOHC\underline{H}_2OCH_2Ph$), 3.37 (dd, J = 8.9, 8.9 Hz, 1H, $CHOHC\underline{H}_2OCH_2Ph$), 3.07 (dq, J = 12.8, 6.4 Hz, 1H, $CH{=}CHC\underline{H}CH_3CHOHAr$), 2.92 (s, 1H, OH), 1.78–1.44 (m, 8H, OH, $CHOHC\underline{H}_2C\underline{H}_2CHOHC\underline{H}_2CHOH$), 0.95 (d, J = 6.8 Hz, 3H, $CH{=}CHCHC\underline{H}_3CHOHAr$);

^{13}C NMR (126 MHz, $CDCl_3$) δ 139.8, 137.9, 133.7, 133.4, 130.9(2C), 128.9(2C), 128.4(2C), 127.8(2C), 127.2, 121.5, 77.7, 74.4, 73.2, 70.3, 68.7, 64.3, 42.1, 38.2, 33.4, 29.8, 17.4;

HRMS: cald. for $C_{25}H_{33}BrO_5$ $(M+Na)^+$ 515.1409; found 515.1397 (TOF MS ES+).

(1*S*,2*R*,5*R*,7*S*,10*S*,*Z*)-11-(benzyloxy)-1-(4-bromophenyl)-2-methylundec-8-ene-1,5,7,10-tetraol (11)

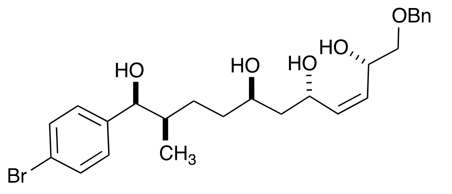

Synthesized by following procedure C

Yield: 26% over 4 reactions (71% avg/rxn)

FTIR (neat): 3365, 3294, 2943, 2872, 2349, 2872, 1631, 1485, 1454, 1070, 1028, 827, 750, 698 cm^{-1};

Optical Rotation: $[\alpha]_D$ = + 5.0 (c = 0.12, $CHCl_3$);

^{1}H NMR (500 MHz, $CDCl_3$) δ 7.50–7.44 (m, 2H, aromatic), 7.39–7.28 (m, 5H, aromatic), 7.22–7.17 (m, 2H, aromatic), 5.71 (ddd, J = 11.5, 7.3, 1.4 Hz, 1H, $CHOHC\underline{H}{=}CHCHOHCH_2OBn$), 5.50 (ddd, J = 11.4, 7.3, 1.3 Hz, 1H, $CHOHCH{=}C\underline{H}CHOHCH_2OBn$), 4.85–4.77 (m, 1H, $C\underline{H}OHCH{=}CHCHOHCH_2OBn$), 4.75–4.69 (m, 1H, $CHOHCH{=}CHC\underline{H}OHCH_2OBn$), 4.62–4.54 (m, 3H, 4-$BrC_6H_4C\underline{H}OHCH(CH_3)CH_2$, $CH_2OC\underline{H}_2Ph$), 3.88 (bs, 1H, OH), 3.76–3.68 (m, 1H, $CH_2C\underline{H}OHCH_2CHOHCH{=}CH$), 3.50 (dd, J = 9.4, 4.1 Hz, 1H, $C\underline{H}_2OBn$), 3.45 (dd, J = 9.4, 7.6 Hz, 1H, $C\underline{H}_2OBn$), 1.84–1.74 (m, 1H, 4-$BrC_6H_4CHOHC\underline{H}(CH_3)CH_2$), 1.72–1.68 (m, 1H, OH), 1.67–1.62 (m, 2H, $CHOHC\underline{H}_2CHOH$), 1.46–1.37 (m, 2H, $CH_2C\underline{H}_2CHOHCH_2CHOH$), 1.26 (s, 4H, OH, $C\underline{H}_2CH_2CHOHCH_2CHOH$), 0.88 (d, J = 6.8 Hz, 3H, 4-$BrC_6H_4CHOHCH(C\underline{H}_3)CH_2$).

^{13}C NMR (126 MHz, $CDCl_3$) δ 142.5, 137.5, 136.5, 131.2 (2C), 129.2, 128.5 (2C), 128.0 (2C), 127.99, 127.9 (2C), 120.9, 76.9, 73.6, 73.5, 67.3, 66.2, 62.9, 42.6, 40.0, 28.9, 28.8, 14.3.

HRMS: cald. for $C_{25}H_{33}BrO_5$ $(M+Na)^+$ 515.1409; found (TOF MS ES+) 515.1398.

(1*S*,2*R*,3*Z*,5*S*,7*S*,8*E*,10*S*)-11-(benzyloxy)-1-(4-bromophenyl)-2-methylundeca-3,8-diene-1,5,7,10-tetraol (12)

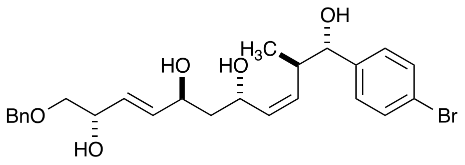

Synthesized by following procedure E

Yield: 38% over 3 reactions (73% avg/rxn)

FTIR (neat): 3377, 3330, 2962, 2926, 2868, 1865, 1591, 1454, 1215, 1101, 1070, 1009, 976 cm^{-1};

Optical Rotation: $[\alpha]_D$ =-38.8 (*c* = 0.45, $CHCl_3$);

^{1}H NMR (500 MHz, $CDCl_3$) δ 7.45 (d, *J* = 8.4 Hz, 2H, aromatic), 7.40–7.29 (m, 5H, aromatic), 7.15 (d, *J* = 8.4 Hz, 2H, aromatic), 5.91 (ddd, *J* = 15.6, 5.6, 1.4 Hz, 1H, CHOHCH=C<u>H</u>CHOHCH$_2$CHOH), 5.74 (ddd, *J* = 15.6, 5.8, 1.4 Hz, 1H, CHOHC<u>H</u>=CHCHOHCH$_2$), 5.63 (ddd, *J* = 10.9, 8.2, 0.9 Hz, 1H, CH$_2$CHOHC<u>H</u>=CHCHCH$_3$), 4.97 (td, *J* = 10.6, 1.2 Hz, 1H, CHOHCH=C<u>H</u>CHCH3), 4.65 (ddd, *J* = 11.4, 6.3, 2.2 Hz, 1H, CH$_2$C<u>H</u>OHCH=CHCHCH$_3$), 4.58 (d, *J* = 2.9 Hz, 4H, OH, PhC<u>H$_2$</u>OCH$_2$CHOHCH=CHCHOHCH$_2$CHOHCH=CHCHCH$_3$C<u>H</u>OHAr), 4.48 (s, 1H PhCH$_2$OCH$_2$CHOHCH=CHC<u>H</u>OHCH$_2$CHOH), 4.40 (s, 1H PhCH$_2$OCH$_2$C<u>H</u>OHCH=CH), 3.56 (dd, *J* = 9.6, 3.3 Hz, 1H, PhCH$_2$OC<u>H$_2$</u>CHOH), 3.38 (dd, *J* = 9.6, 8.1 Hz, 1H, PhCH$_2$OC<u>H$_2$</u>CHOH), 3.13 (s, 1H, OH), 3.12–3.01 (m, 1H, CHOHCH=CHC<u>H</u>CH$_3$), 2.62 (s, 1H, OH), 1.87 (ddd, *J* = 14.4, 8.7, 3.6 Hz, 1H, CH=CHCHOHC<u>H$_2$</u>CHOH), 1.70 (ddd, *J* = 14.3, 7.5, 3.3 Hz, 1H, CH=CHCHOHC<u>H$_2$</u>CHOH), 1.61 (s, 1H, OH), 0.9 (s, 3H, CH=CHCHC<u>H$_3$</u>);

^{13}C NMR (126 MHz, $CDCl_3$) δ 139.6, 137.7, 134.8, 133.8, 133.5, 130.9 (2C), 128.9 (2C), 128.5 (2C), 128.4, 127.90, 127.8 (2C), 121.6, 76.9, 74.1, 73.4, 70.7, 69.5, 64.1, 41.9, 38.2, 17.4;

HRMS: cald. for $C_{25}H_{31}BrO_5$ $(M+Na)^+$513.1253; found 513.1255 (TOF MS ES+).

(1*S*,2*R*,3*E*,5*S*,7*S*,8*Z*,10*S*)-11-(benzyloxy)-1-(4-bromophenyl)-2-methylundeca-3,8-diene-1,5,7,10-tetraol (13)

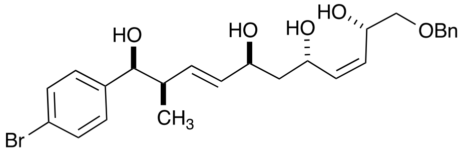

Synthesized by following procedure D

Yield: 35% over 3 reactions (70% avg/rxn)

FTIR (neat): 3440, 3417, 3386, 2390, 1643, 1633, 1054, 698, 522 cm^{-1};

Optical Rotation: $[\alpha]_D$ = - 4.81 (*c* = 0.22, $CHCl_3$)

^{1}H NMR (500 MHz, $CDCl_3$) δ 7.47–7.43 (m, 2H, aromatic), 7.37–7.29 (m, 5H, aromatic), 7.18–7.12 (m, 2H, aromatic), 5.68 (ddd, *J* = 11.4, 7.6, 1.4 Hz, 1H, $CHOHC\underline{H}=CH_{cis}CHOHCH_2OBn$), 5.61 (ddd, *J* = 15.6, 7.0, 1.0 Hz, 1H, $C\underline{H}=CH_{trans}CHOHCH_2CHOH$), 5.53 (d, *J* = 6.2 Hz, 1H, $CH=C\underline{H}_{trans}CHOHCH_2CHOH$), 5.48 (ddd, *J* = 11.4, 7.4, 1.3 Hz, 1H, $CH=CH_{cis}C\underline{H}OHCH_2CHOH$), 4.76 (dd, *J* = 7.9, 2.9 Hz, 1H, $C\underline{H}OHCH=CHCHOHCH_2OBn$), 4.68 (ddd, *J* = 7.5, 4.1, 1.4 Hz, 1H, $CHOHCH=CHC\underline{H}OHCH_2OBn$), 4.59 (d, *J* = 5.1 Hz, 1H, 4-$BrC_6H_4C\underline{H}OHCH(CH_3)CH_2$), 4.56 (bs, 2H, $C\underline{H}_2OBn$), 4.35 (dd, *J* = 6.6, 10.4 Hz, 1H, $CH=CHC\underline{H}OHCH_2CHOH$), 3.55 (s, 1H, OH), 3.48 (dd, *J* = 9.5, 4.1 Hz, 1H, $C\underline{H}_2OBn$), 3.44 (dd, *J* = 9.5, 7.5 Hz, 1H, $C\underline{H}_2OBn$), 3.16 (s, 1H, OH), 2.90 (s, 1H, OH), 2.52 (dd, *J* = 12.6, 6.7 Hz, 1H, 4-$BrC_6H_4CHOHC\underline{H}(CH_3)CH_2$), 2.46 (s, 1H, OH), 1.74 (ddd, *J* = 14.4, 8.7, 3.8 Hz, 1H, $CHOHC\underline{H}_2CHOH$), 1.61 (ddd, *J* = 14.3, 7.5, 3.3 Hz, 1H, $CHOHC\underline{H}_2CHOH$), 0.96 (d, *J* = 6.8 Hz, 3H, 4-$BrC_6H_4CHOHCH(C\underline{H}_3)CH_2$)

^{13}C NMR (126 MHz, $CDCl_3$) δ 141.6, 137.5, 136.3, 133.8, 132.5, 131.1 (2C), 129.2, 128.5 (2C), 128.1 (2C), 127.9, 127.9 (2C), 121.0, 76.6, 73.6, 73.5, 70.1, 67.2, 65.7, 43.3, 42.8, 14.0.

HRMS: cald. for $C_{25}H_{31}BrO_5$ $(M+Na)^+$513.1253; found (TOF MS ES+) 513.1237.

(1*S*,2*R*,5*R*,7*R*,10*S*)-11-(benzyloxy)-1-(4-bromophenyl)-2-methylundecane-1,5,7,10-tetraol (14)

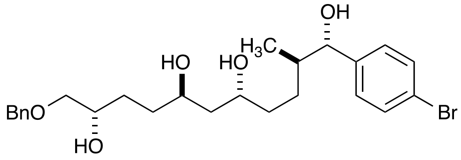

Synthesized by following procedure F

Yield: 26% over 4 reactions (72% avg/rxn)

FTIR (neat): 3377, 3330, 2962, 2926, 2868, 1865, 1454, 1215, 1070, 1009, 976 cm^{-1};

Optical Rotation: $[\alpha]_D$ = -11.4 (*c* = 0.285, $CHCl_3$);

^{1}H NMR (500 MHz, $CDCl_3$) δ 7.46 (d, *J* = 8.3 Hz, 2H, aromatic), 7.38–7.28 (m, 5H, aromatic), 7.2 (d, *J* = 8.3 Hz, 2H, aromatic), 4.60 (d, *J* = 4.9 Hz, 1H, $CH_2CHCH_3C\underline{H}OHAr$), 4.56 (s, 2H, $PhC\underline{H}_2OCH_2CHOH$), 4.06–3.93 (m, 1H, $CH_2C\underline{H}OHCH_2CHOH$), 3.92–3.85 (m, 2H, $PhCH_2OCH_2C\underline{H}OHCH_2CH_2CHOHCH_2C\underline{H}OH$), 3.50 (dd, *J* = 9.3, 3.5 Hz, 1H, $PhCH_2OC\underline{H}_2CHOH$), 3.37 (m, 1H, $PhCH_2OC\underline{H}_2CHOH$), 1.84–1.74 (m, 1H $CH_2C\underline{H}CH_3CHOHAr$), 1.65–1.55 (m, 11H, OH, aliphatic), 1.46–1.37 (m, 2H, aliphatic), 1.28–1.21 (m, 1H, aliphatic), 0.95 (d, *J* = 6.8 Hz, 3H, $CH_2CH_2CHC\underline{H}_3CHOHAr$);

^{13}C NMR (126 MHz, $CDCl_3$) δ 142.6, 137.7, 131.2, 128.5(2C), 128.1(2C), 127.8(2C), 126.4(2C), 121.0, 77.1, 74.3, 73.4, 70.3, 69.4, 69.0, 42.4, 40.0, 34.8, 33.4, 29.0, 28.9, 14.3;

HRMS: cald. for $C_{25}H_{35}BrO_5$ $(M+Na)^+$ 517.1566; found 517.1584 (TOF MS ES+).

(1*S*,3*S*,7*S*,9*R*,*Z*)-9-((*S*)-4-benzyloxy-3-hydroxybutyl)-3-((benzyloxy)methyl)-2,10,11-trioxa-1-phosphabicyclo[5.3.1]undec-5-ene 1-oxide (15)

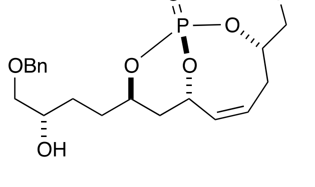

Synthesized by following procedure B

Yield: 40% over 3 reactions (72% avg/rxn)

FTIR (neat): 3413, 3028, 2923, 2858, 1679, 1452, 1363, 1288, 1101, 979 cm^{-1};

Optical Rotation: $[\alpha]_D$ = +7.6 (*c* = 0.41, $CHCl_3$);

^{1}H NMR (500 MHz, $CDCl_3$) δ 7.40–7.30 (m, 10H, aromatic), 5.69 (dtd, *J* = 11.8, 8.8, 2.9 Hz, 1H, $CHOPCH{=}C\underline{H}CH_2CHOP$), 5.45 (dt, *J* = 11.8, 1.7 Hz, 1H, $CHOPC\underline{H}{=}CHCH_2CHOP$), 5.25 (dd, *J* = 25.0, 4.3 Hz, 1H, $CH_2C\underline{H}OPCH_2CHOPCH{=}CH$), 4.76–4.65 (m, 1H, $CH{=}CHCH_2C\underline{H}OPCH_2OBn$), 4.59–4.52 (m, 5H, $OC\underline{H}_2Ph$, $CHOPCH_2C\underline{H}OPCH{=}CH$), 3.79 (ddd, *J* = 11.4, 8.9, 3.1Hz, 1H, $PhCH_2OCH_2C\underline{H}OHCH_2$), 3.51 (ddd, *J* = 11.3, 9.7, 4.2 Hz, 2H, $PhCH_2OC\underline{H}_2CHOHC_2H_4CHOPCH_2CHOPCH{=}CHCH_2CHOPC\underline{H}_2OCH_2Ph$), 3.43 (dd, *J* = 9.4, 7.4 Hz, 1H, $CH{=}CHCH_2CHOPC\underline{H}_2OBn$), 3.36–3.29 (m, 2H, $BnOC\underline{H}_2CHOHC_2H_4CHOPCH_2CHOPCH{=}CHC\underline{H}_2CHOP$), 2.36 (dd, *J* = 13.9, 8.5 Hz, 1H, $CH{=}CHC\underline{H}_2CHOPCH_2OBn$), 2.14 (ddd, *J* = 14.5, 11.7, 6.0 Hz, 1H, $CHOPC\underline{H}_2CHOPCH{=}CH$), 1.91–1.79 (m, 1H, $CHOHCH_2C\underline{H}_2CHOPCH_2CHOP$), 1.78–1.65 (m, 3H, $CHOHC\underline{H}_2C\underline{H}_2CHOPC\underline{H}_2CHOP$), 1.62 (s, 1H, OH), 1.44 (dddd, *J* = 15.2, 12.9, 7.5, 3.0 Hz, 1H, $CHOHC\underline{H}_2CH_2CHOPCH_2CHOP$);

^{13}C NMR (126 MHz, $CDCl_3$) δ 137.8, 137.7, 131.5, 128.5 (2C), 128.5 (2C), 127.9, 127.8, 127.7 (2C), 127.6 (2C), 125.03, 78.4 (d, J_{CP} = 7.1 Hz), 78.1 (d, J_{CP} = 7.2 Hz) 74.4, 73.4, 73.2, 72.3 (d, J_{CP} = 4.9 Hz), 70.4 (d, J_{CP} = 15.5 Hz), 70.2, 36.2 (d, J_{CP} = 6.5 Hz), 32.4 (d, J_{CP} = 8.8 Hz), 28.5, 27.4;

^{31}P NMR (162 MHz, $CDCl_3$) δ –8.2;

HRMS: cald. for $C_{26}H_{33}O_7P$ $(M+Na)^+$ 511.1862; found 511.1855 (TOF MS ES+).

(1*S*,3*S*,6*S*,8*R*)-8-((*S*)-5-benzyloxy-4-hydroxypentyl)-3-((benzyloxy)methyl)-2,9,10-trioxa-1-phosphabicyclo[4.3.1]dec-4-ene 1-oxide (16)

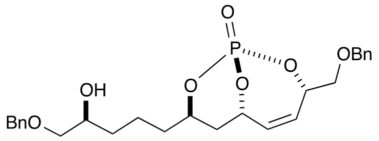

Synthesized by following procedure A

Yield: 35% over 3 reactions (70% avg/rxn)

FTIR (neat): 3520, 3444, 2395, 1633, 1286, 1101, 973, 734 cm^{-1};

Optical Rotation: $[\alpha]_D$ = + 21.57(*c* = 0.26, $CHCl_3$);

^{1}H NMR (500 MHz, $CDCl_3$) δ 7.42–7.28 (m, 10H, aromatic), 6.02 (ddd, *J* = 11.9, 3.0, 2.1 Hz, 1H, $CH{=}C\underline{H}CHOPCH_2OBn$), 5.57 (ddd, *J* = 11.9, 3.9, 2.4 Hz, 1H, $C\underline{H}{=}CHCHOPCH_2OBn$), 5.28 (ddddd, *J* = 5.3, 5.3, 2.7, 2.7, 2.7 Hz, 1H, $CH{=}CHC\underline{H}OPCH_2OBn$), 5.19 (ddddd, J_{PH}= 24.5, J_{HH}= 6.2, 4.1, 1.8, 1.8 Hz, 1H, $CH_2C\underline{H}OPCH{=}CH$), 4.66–4.52 (m, 5H, $CH_2OC\underline{H}_2Ph$, $CH_2CH_2CH_2C\underline{H}OP$), 3.84–3.77 (m, 1H, $CH_2C\underline{H}OHCH_2OBn$), 3.72 (ddd, *J* = 10.2, 5.1, 1.1 Hz, 1H, $C\underline{H}_2OBn$), 3.61 (dd, *J* = 10.2, 6.1 Hz, 1H, $C\underline{H}_2OBn$), 3.50 (dd, *J* = 9.4, 3.1 Hz, 1H, $C\underline{H}_2OBn$), 3.32 (dd, *J* = 9.4, 7.9 Hz, 1H, $C\underline{H}_2OBn$), 2.34 (d, *J* = 3.4 Hz, 1H, $BnOCH_2CHO\underline{H}CH_2CH_2CH_2CHOP$), 2.22–2.12 (m, 2H, $CHOPC\underline{H}_2CHOP$), 1.83–1.55 (m, 4H, $BnOCH_2CHOHCH_2C\underline{H}_2C\underline{H}_2CHOP$), 1.52–1.37 (m, 2H, $BnOCH_2CHOHC\underline{H}_2CH_2CH_2CHOP$).

^{13}C NMR (126 MHz, $CDCl_3$) δ 137.8, 137.5, 129.9, 129.6, 128.5 (4C), 127.9, 127.8, 127.7 (4C), 77.3 (d, J_{CP} = 6.7 Hz), 76.6 (d, J_{CP} = 6.8 Hz), 74.4, 73.5, 73.4, 72.1 (d, J_{CP} = 6.0 Hz), 71.2 (d, J_{CP} = 12.2 Hz), 70.0, 35.4 (d, J_{CP} = 9.3 Hz), 34.7 (d, J_{CP} = 6.0 Hz), 32.4, 20.5.

^{31}P NMR (162 MHz, $CDCl_3$) δ -5.46 ;

HRMS: cald. for $C_{26}H_{33}O_7PNa$ $(M+Na)^+$ 511. 1862; found (TOF MS ES+) 511.1847.

(2*S*,5*R*,7*S*,11*S*,*Z*)-1,12-bis(benzyloxy)dodec-8-ene-2,5,7,11-tetraol (17)

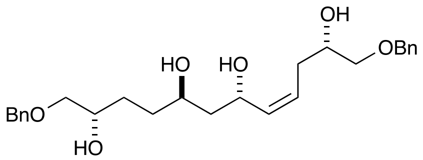

Synthesized by following procedure C

Yield: 24% over 4 reactions (70% avg/rxn)

FTIR (neat): 3367, 2914, 2858, 2914,2331, 1093, 1076, 698 cm^{-1};

Optical Rotation: $[\alpha]_D$ = + 6.8 (c = 0.44, $CHCl_3$);

^{1}H NMR (500 MHz, $CDCl_3$) δ 7.40–7.30 (m, 10H, aromatic), 5.70 (ddt, J = 11.0, 8.2, 1.4 Hz, 1H, CHOHCH=CHCH$_2$CHOH), 5.51 (dddd, J = 11.0, 8.6, 7.5, 1.1 Hz, 1H, CHOHCH=CHCH$_2$CHOH), 4.79 (dt, J = 7.6, 5.6 Hz, 1H, CHOHCH$_2$CHOHCH=CHCH$_2$CHOH), 4.60–4.53 (m, 4H, CH$_2$OCH$_2$Ph), 4.01–3.93 (m, 1H, CH$_2$CHOCH$_2$CHOCH=CH), 3.93 (ddd, J = 11.2, 5.1, 3.5 Hz 1H, CH=CHCH$_2$CHOHCH$_2$OBn), 3.89–3.82 (m, 1H, CH$_2$CH$_2$CHOHCH$_2$OBn), 3.51(td, J = 9.4, 3.3 Hz, 2H, CH=CHCH$_2$CHOHCH$_2$OBn), 3.45–3.33 (m, 2H, CH$_2$CH$_2$CHOHCH$_2$OBn), 3.15 (bs, 1H, OH), 2.90 (bs, 2H, OH), 2.43 (dddd, J = 14.5, 8.6, 5.1, 1.1 Hz, 1H, CH=CHCH$_2$CHOHCH$_2$OBn), 2.30 (dddd, J = 14.2, 7.7, 6.5, 1.3 Hz, 1H, CH=CHCH$_2$CHOHCH$_2$OBn), 1.77–1.67 (m, 2H, CHOHCH$_2$CHOHCH=CHCH$_2$CHOH), 1.67–1.49 (m, 4H, CHOHCH$_2$CH$_2$CHOHCH$_2$CHOH), 1.30 (s, 1H, OH);

^{13}C NMR (126 MHz, $CDCl_3$) δ 137.9, 137.7, 135.6, 128.5 (2C), 128.4 (2C), 127.9, 127.8 (3C), 127.7 (2C), 126.4, 74.4, 73.6, 73.5, 73.3, 70.4, 69.6, 68.9, 65.3, 42.5, 33.5, 31.2, 29.2;

HRMS: cald. for $C_{26}H_{36}O_6$ $(M+Na)^+$ 467.2410; found 467.2390 (TOF MS ES+).

(2*S*,5*S*,7*R*,11*S*,*Z*)-1,12-bis(benzyloxy)dodec-3-ene-2,5,7,11-tetraol (18)

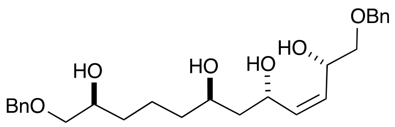

Synthesized by following procedure C

Yield: 23% over 4 reactions (69% avg/rxn)

FTIR (neat): 3400, 3290, 2943, 2350, 1631, 1480, 1070, 750, 698cm^{-1};

Optical Rotation: $[\alpha]_D$ = + 6.32(c = 0.19, $CHCl_3$);

^{1}H NMR (500 MHz, $CDCl_3$) δ 7.40–7.28 (m, 10H, aromatic), 5.70 (ddd, J = 11.5, 7.5, 1.4 Hz, 1H, CHOHCH=CHCHOHCH$_2$OBn), 5.48 (ddd, J = 11.4, 7.4, 1.4 Hz, 1H, CHOHCH=CHCHOHCH$_2$OBn), 4.80 (ddd, J = 10.4, 7.3, 3.4 Hz, 1H, CHOHCH=CHCHOHCH$_2$OBn), 4.75–4.67 (m, 1H, CHOHCH=CHCHOHCH$_2$OBn), 4.59–4.53 (m, 4H, CH$_2$OCH$_2$Ph), 3.97–3.88 (m, 1H, CH$_2$CHOHCH$_2$CHOHCH=CH), 3.88–3.79 (m, 1H,

$BnOCH_2C\underline{H}OHCH_2CH_2CH_2$), 3.56–3.41 (m, 4H, $C\underline{H}_2OBn$), 3.40–3.29 (m, 2H, $BnOC\underline{H}_2CHOHCH_2CH_2CH_2$), 1.66 (ddd, J = 14.5, 7.5, 2.6 Hz, 1H, $CHOHC\underline{H}_2CHOH$), 1.63–1.57 (m, 3H, $CHOHC\underline{H}_2CHOH$, $BnOCH_2CHOHC\underline{H}_2CH_2CH_2$, $O\underline{H}$), 1.53–1.35 (m, 6H, $BnOCH_2CHOHC\underline{H}_2C\underline{H}_2C\underline{H}_2CHOH$).

^{13}C NMR (126 MHz, $CDCl_3$) δ 137.9, 137.6, 136.5, 129.2, 128.5 (2C), 128.46 (2C), 127.9, 127.85 (2C), 127.8, 127.7 (2C), 74.5, 73.6, 73.4, 73.3, 70.23, 68.8, 67.1, 66.1, 42.7, 37.2, 32.8, 21.5.

HRMS: cald. for $C_{26}H_{36}O_6$ $(M+Na)^+$ 467.2410; found (TOF MS ES+) 467.2413.

(2*S*,3*E*,5*S*,7*S*,8*Z*,11*S*)-1,12-bis(benzyloxy)dodeca-3,8-diene-2,5,7,11-tetraol (19)

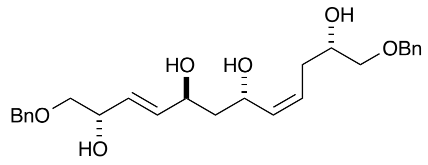

Synthesized by following procedure E

Yield: 42% over 3 reactions (75% avg/rxn)

FTIR (neat): 3377, 3361, 3028, 2916, 2858, 1602, 1452, 1101 cm^{-1};

Optical Rotation: $[\alpha]_D$ = +10.4 (c = 0.67, $CHCl_3$);

^{1}H NMR (500 MHz, $CDCl_3$) δ 7.40–7.29 (m, 10H, aromatic), 5.89 (ddd, J = 15.5, 5.4, 1.3 Hz, 1H, $CHOHCH{=}C\underline{H}CHOHCH_2$), 5.77–5.64 (m, 2H, $CHOHC\underline{H}{=}CHCHOHCH_2CHOHC\underline{H}{=}CHCH_2$), 5.53–5.48 (m, 1H, $CHOHCH{=}C\underline{H}CH_2CHOHCH_2OBn$), 4.71 (td, J = 2.1 Hz, 1H, $CHOHCH_2C\underline{H}OHCH{=}CHCH_2$), 4.57 (d, J = 2.1 Hz, 2H, $CH_2OC\underline{H}_2Ph$), 4.55 (d, J = 3.8 Hz, 2H, $CH_2OC\underline{H}_2Ph$), 4.46 (bs, 1H, $CHOHCH{=}CHC\underline{H}OHCH_2$), 4.38 (bs, 1H, $PhCH_2OCH_2C\underline{H}OHCH{=}CHCHOH$), 3.91 (ddt, J = 11.0, 6.7, 3.4 Hz, 1H, $CH{=}CHCH_2C\underline{H}OHCH_2OBn$), 3.54 (ddd, J = 9.5, 3.4, 2.2 Hz, 1H, $CH{=}CHCHOHC\underline{H}_2OBn$), 3.51 (dd, J = 9.5, 3.4 Hz, 1H, $CH{=}CHCH_2CHOHC\underline{H}_2OBn$), 3.45–3.32 (m, 2H, $C\underline{H}_2OBn$), 3.06 (s, 1H, OH), 2.82 (s, 1H, OH), 2.60 (s, 1H, OH) 2.42 (dddd, J = 13.9, 8.7, 5.1, 1.3 Hz, 1H, $CH{=}CHC\underline{H}_2CHOHCH_2OBn$), 2.28 (dddd, J = 14.1, 7.6, 6.3, 1.3 Hz, 1H, $CH{=}CHC\underline{H}_2CHOHCH_2OBn$), 1.85 (ddd, J = 14.3, 8.1, 3.6 Hz, 1H, $CHOHC\underline{H}_2CHOHCH{=}CHCH_2$), 1.70 (ddd, J = 14.3, 7.6, 3.7 Hz, 1H, $CHOHC\underline{H}_2CHOHCH{=}CHCH_2$), 1.64 (s, 1H, OH);

^{13}C NMR (126 MHz, $CDCl_3$) δ 137.7, 137.6, 135.4, 134.9, 128.51 (2C), 128.5, 128.48 (2C), 128.4, 127.92, 127.89, 127.86, 127.8 (2C), 126.6, 74.1, 73.5, 73.4, 73.36, 70.7, 69.6, 69.5, 64.9, 42.4, 31.1;

HRMS: cald. for $C_{26}H_{34}O_6$ $(M+Na)^+$ 465.2253; found 465.2241 (TOF MS ES+).

(2*S*,3*Z*,5*S*,7*S*,8*E*,11*S*)-1,12-bis(benzyloxy)dodeca-3,8-diene-2,5,7,11-tetraol (20)

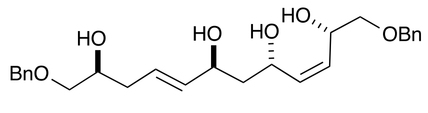

Synthesized by following procedure D

Yield: 40% over 3 reactions (72% avg/rxn)

FTIR (neat): 3408, 3402, 3385, 2918, 2852, 2349, 1637, 1632, 1072, 1027, 972, 737, 698 cm^{-1};

Optical Rotation: $[\alpha]_D$ = + 5.92(c = 0.14, $CHCl_3$);

^{1}H NMR (500 MHz, $CDCl_3$) δ 7.42–7.28 (m, 10H, aromatic), 5.75–5.64 (m, 2H, $CHOHC\underline{H}=C\underline{H}CHOHCH_2OBn$), 5.62–5.53 (m, 1H, $BnOCH_2CHOHCH_2CH=C\underline{H}CHOH$), 5.48 (ddd, *J* = 11.3, 7.4, 1.3 Hz, 1H, $BnOCH_2CHOHCH_2C\underline{H}=CHCHOH$), 4.78 (ddd, *J* = 7.8, 7.8, 3.0 Hz, 1H, $C\underline{H}OHCH=CHCHOHCH_2OBn$), 4.70 (dddd, *J* = 7.5, 7.5, 4.3, 1.4 Hz, 1H, $CHOHCH_2CHOHCH=CHC\underline{H}OHCH_2OBn$), 4.59–4.52 (m, 4H, $CH_2OC\underline{H}_2Ph$), 4.42–4.35 (m, 1H, $CHOHCH_2CH=CHC\underline{H}OH$), 3.90–3.82 (m, 1H, $BnOCH_2C\underline{H}OHCH_2CH=CH$), 3.54–3.40 (m, 4H, $BnOC\underline{H}_2CHOH$, OH), 3.36 (dd, *J* = 9.5, 7.3 Hz, 2H, $BnOC\underline{H}_2CHOH$), 2.30–2.13 (m, 4H, $BnOCH_2CHOHC\underline{H}_2CH=CH$), 1.77 (ddd, *J* = 14.3, 8.6, 3.6 Hz, 1H, $CHOHC\underline{H}_2CHOH$), 1.64 (ddd, *J* = 14.4, 7.7, 3.3 Hz, 1H, $CHOHC\underline{H}_2CHOH$).

^{13}C NMR (126 MHz, $CDCl_3$) δ 137.8, 137.6, 136.3, 135.5, 129.3, 128.5 (2C), 128.4 (2C), 127.9, 127.85 (2C), 127.8, 127.7 (2C), 126.7, 77.2, 73.9, 73.6, 73.4, 73.3, 69.9, 67.1, 65.6, 42.6, 36.2;

HRMS: cald. for $C_{26}H_{34}O_6$ $(M+Na)^+$ 465.2253; found (TOF MS ES+) 465.2236.

(2*S*,5*R*,7*R*,11*S*)-1,12-bis(benzyloxy)dodecane-2,5,7,11-tetraol (21)

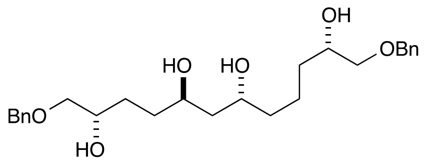

Synthesized by following procedure F

Yield: 34% over 4 reactions (77% avg/rxn)

FTIR (neat): 3283, 2943, 2394, 1454, 1093, 1076, 737 cm^{-1};

Optical Rotation: $[\alpha]_D$ = + 2.80 (*c* = 0.25, $CHCl_3$);

^{1}H NMR (500 MHz, $CDCl_3$) δ 7.40–7.29 (m, 10H, aromatic), 4.60–4.52 (m, 4H, $CH_2OC\underline{H}_2Ph$), 4.04–3.91 (m, 2H, $C\underline{H}OHCH_2C\underline{H}OH$), 3.92–3.76 (m, 2H, $C\underline{H}OHCH_2OBn$), 3.51 (ddd, *J* = 9.5, 4.9, 3.2 Hz, 2H, $BnOC\underline{H}_2CHOH$), 3.36 (ddd, *J* = 17.2, 9.4, 8.0 Hz, 2H, $BnOC\underline{H}_2CHOH$), 2.80 (s, 1H, OH), 2.61 (s, 1H, OH), 2.40 (bs, 1H, OH), 1.70–1.37 (m, 13H, aliphatic, OH).

^{13}C NMR (126 MHz, $CDCl_3$) δ 137.9, 137.8, 128.5 (4C), 127.9, 127.8, 127.77 (2C), 127.75 (2C), 74.3, 74.5, 73.3 (2C), 70.4, 70.3, 69.3, 69.0, 42.3, 37.2, 33.5, 32.8, 29.1, 21.6.

HRMS: cald. for $C_{26}H_{38}O_6$ $(M+Na)^+$ 469.2566; found (TOF MS ES+) 469.2567.

(4*S*,6*S*)-2-(((1*S*,2*R*)-1-(4-bromophenyl)-2-methylbut-3-en-1-yl)oxy)-4,6-divinyl-1,3,2-dioxaphosphinane 2-oxide (5)

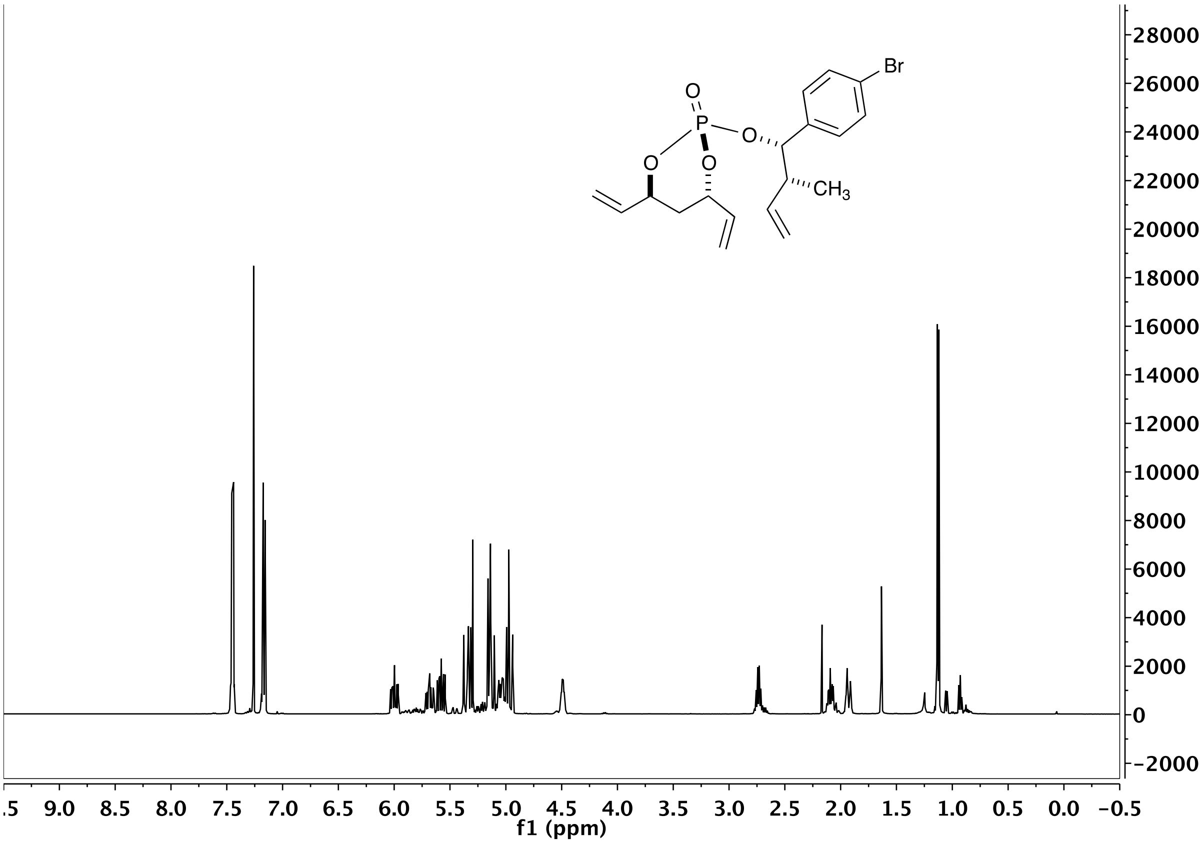

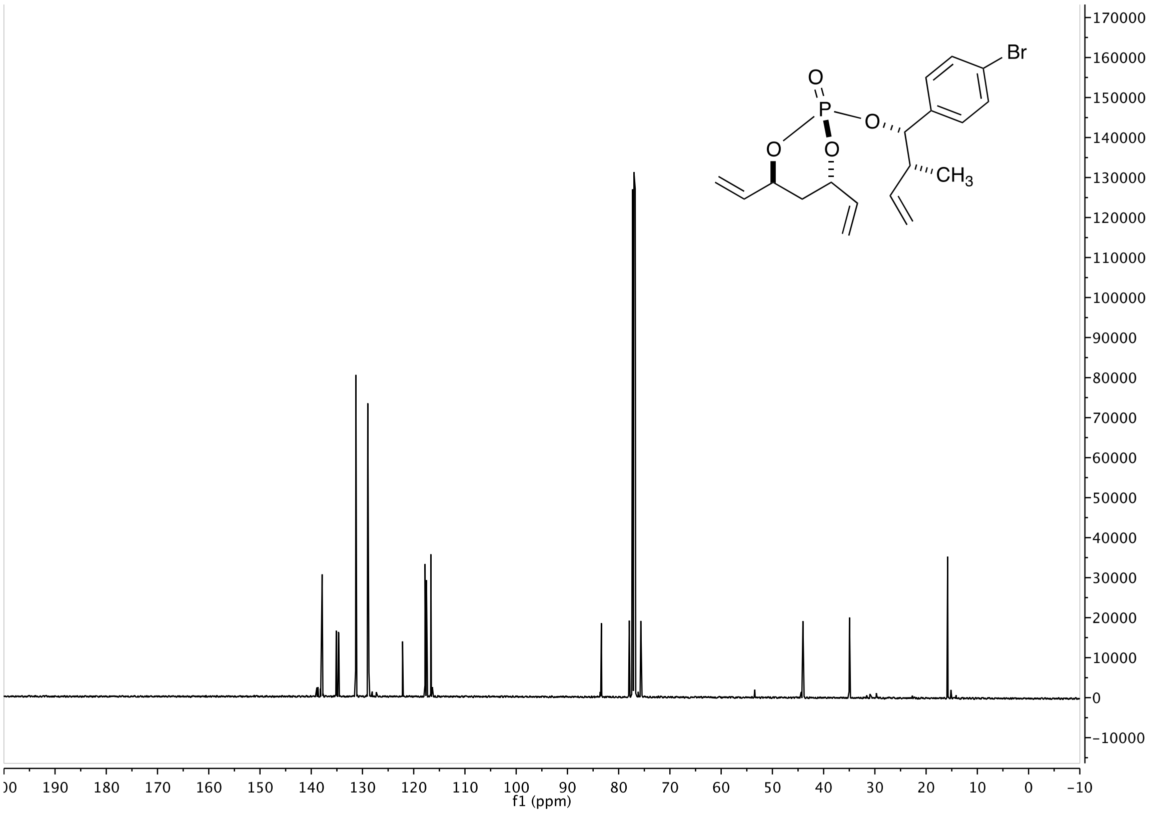

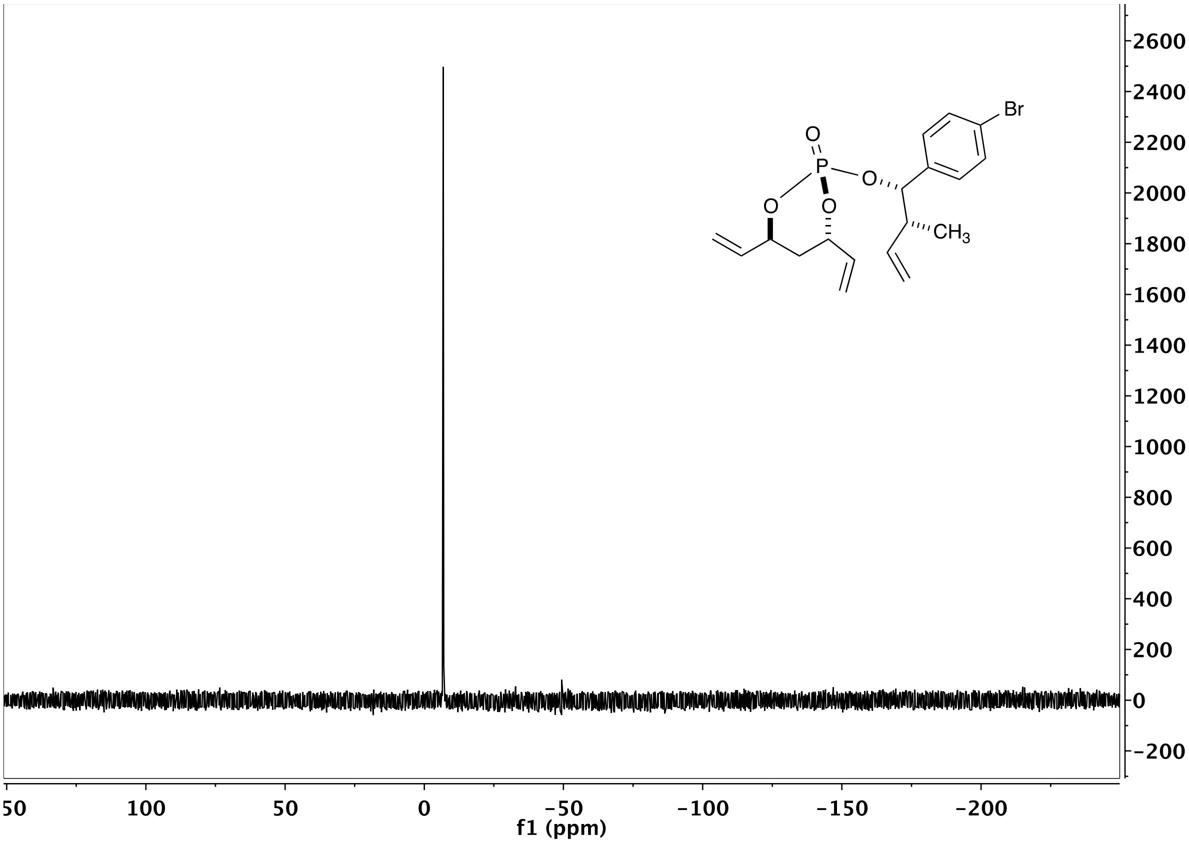

(1*S*,3*S*,4*R*,7*S*,9*R*,*Z*)-9-((*S*)-4-(benzyloxy)-3-hydroxybutyl)-3-(4-bromophenyl)-4-methyl-2,10,11-trioxa-1-phosphabicyclo[5.3.1]undec-5-ene 1-oxide (8)

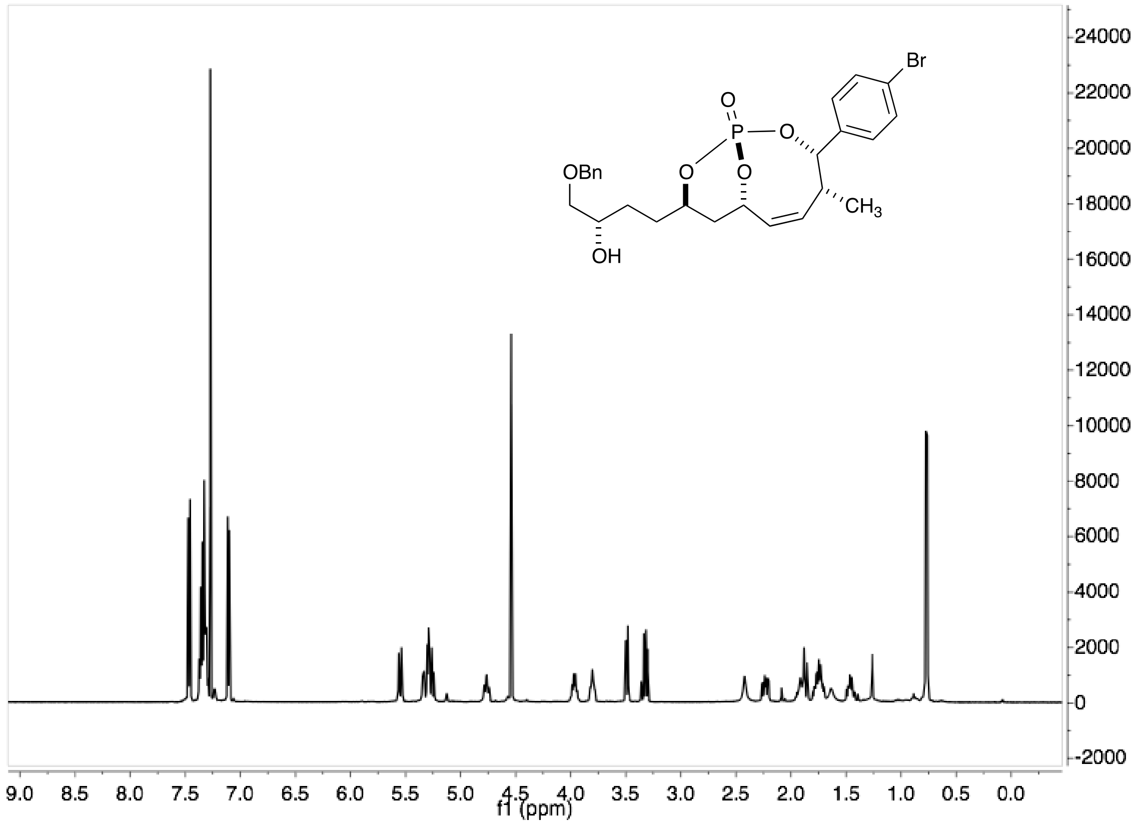

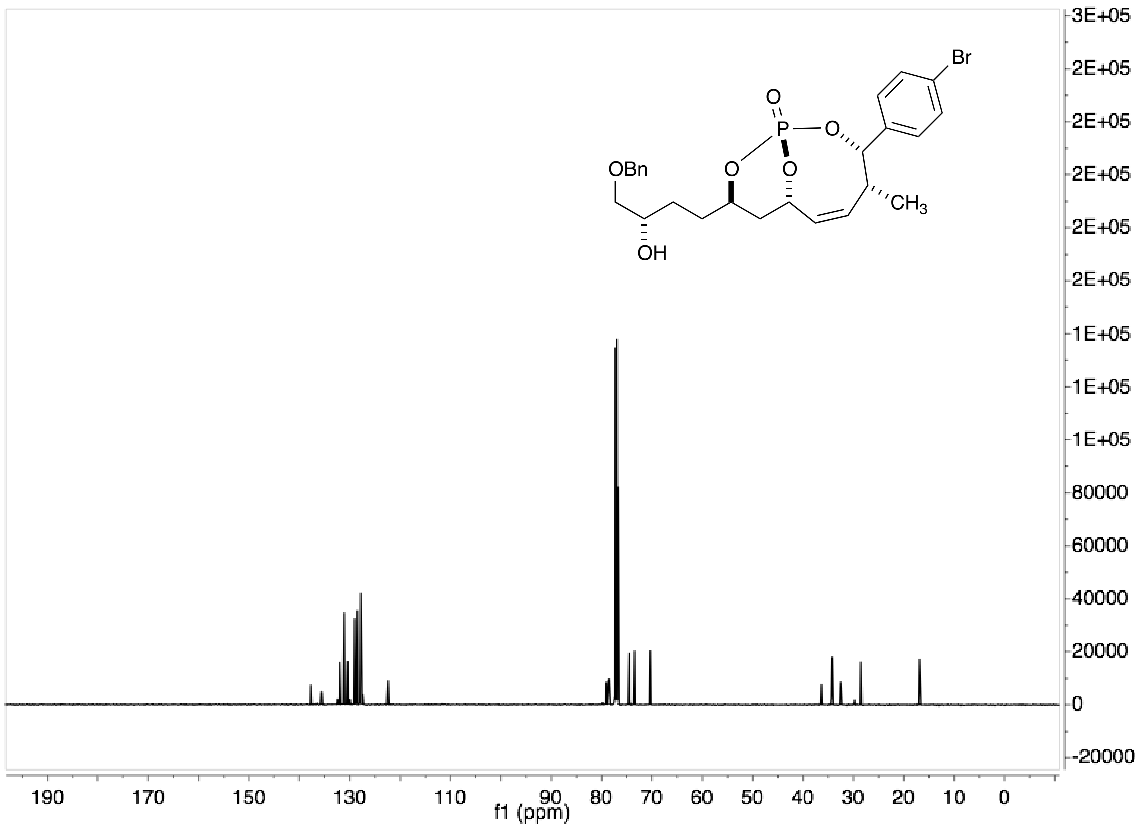
OBn
OH
O
P
O
O
O
Br
CH3
3E+05
2E+05
1E+05
80000
60000
40000
20000
0
-20000
190
170
150
130
110
90
80
70
60
50
40
30
20
10
0
f1 (ppm)

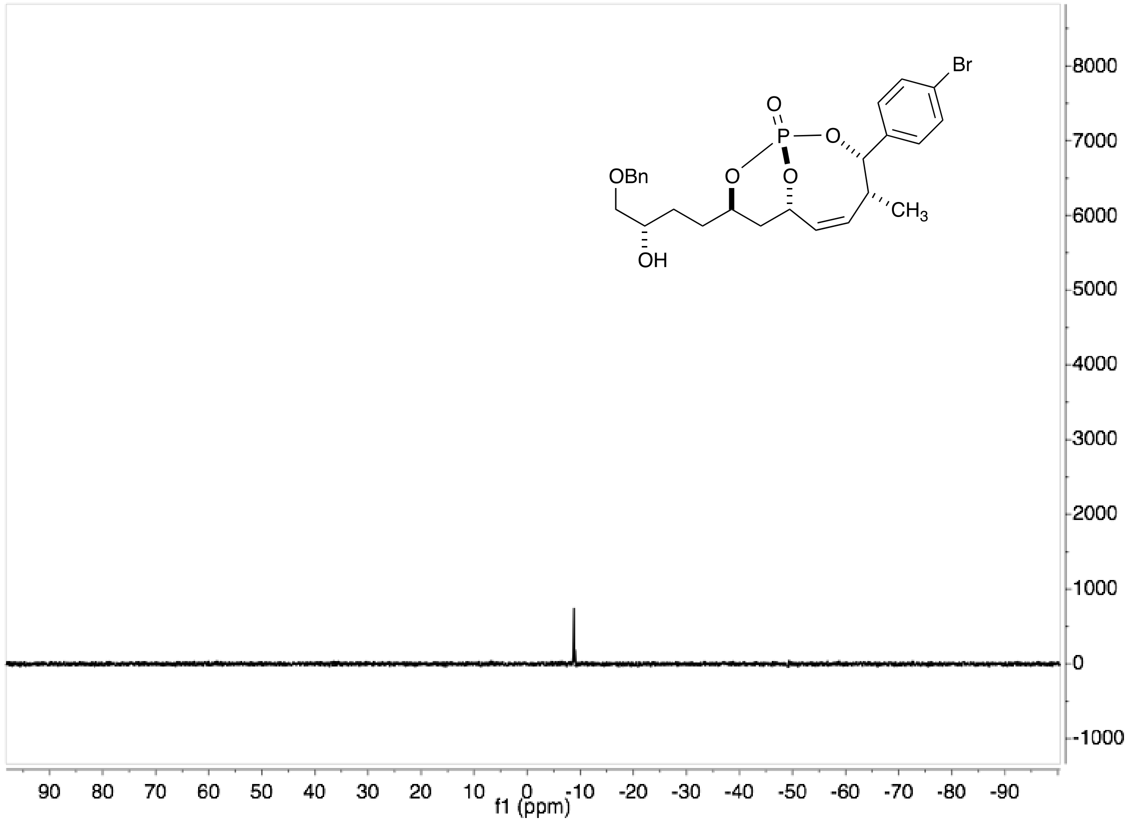
OBn
OH
O
P
O
O
O
Br
CH3
8000
7000
6000
5000
4000
3000
2000
1000
0
-1000
90
80
70
60
50
40
30
20
10
0
-10
-20
-30
-40
-50
-60
-70
-80
-90
f1 (ppm)

(1*S*,3*S*,6*S*,8*R*)-3-((benzyloxy)methyl)-8-((3*R*,4*S*)-4-(4-bromophenyl)-4-hydroxy-3-methylbutyl)-2,9,10-trioxa-1-phosphabicyclo[4.3.1]dec-4-ene 1-oxide (9)

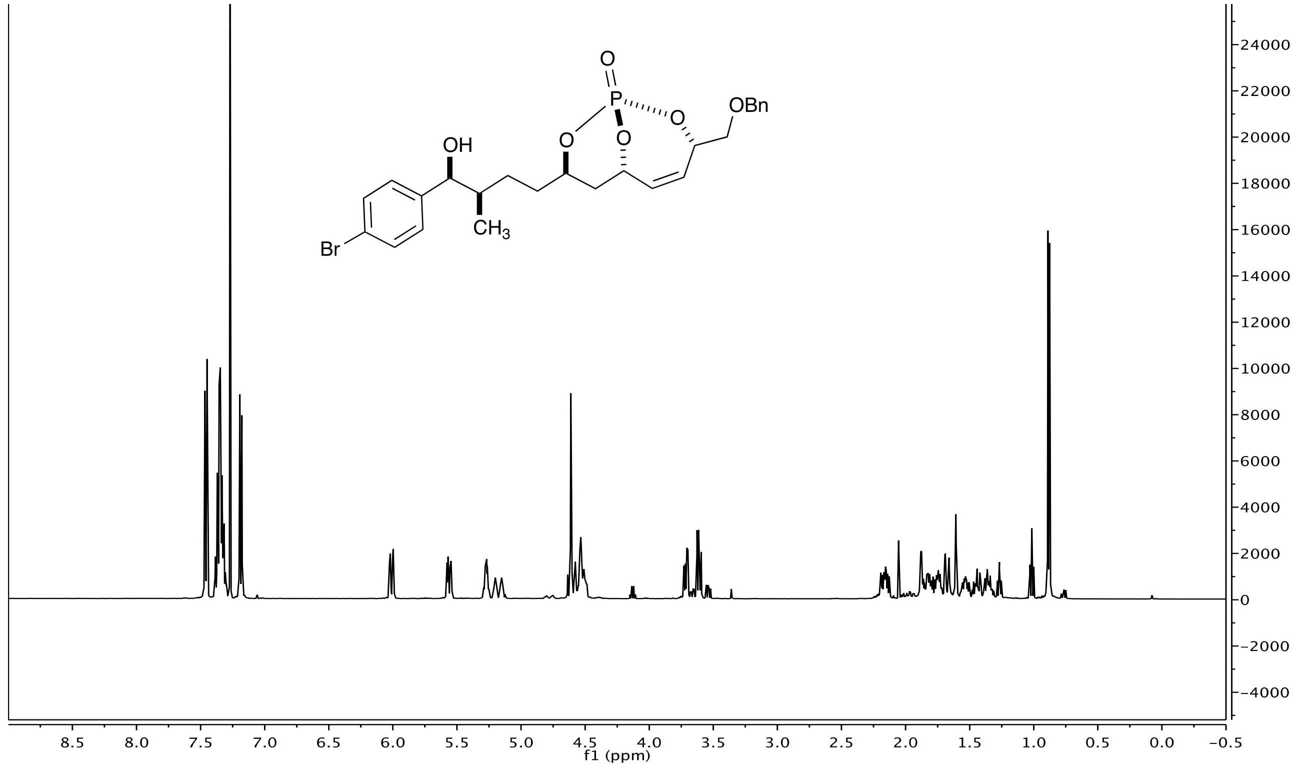

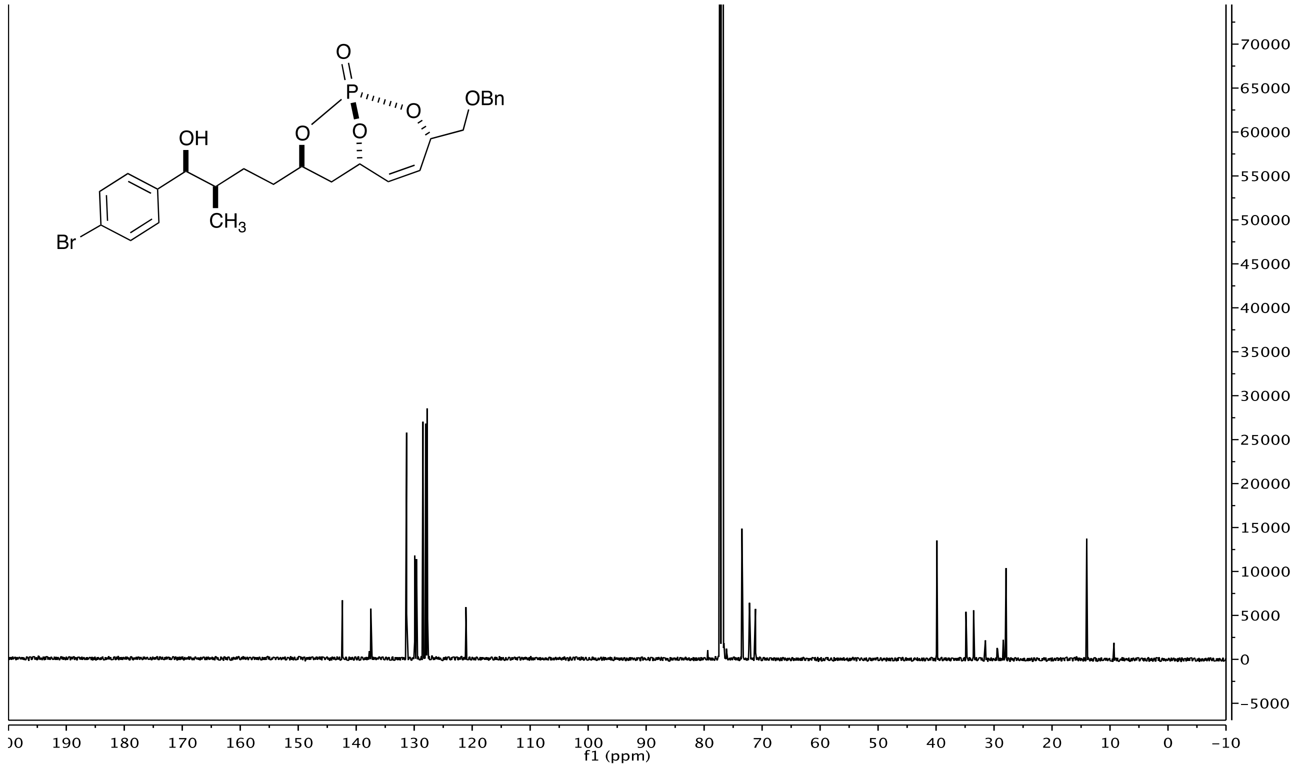

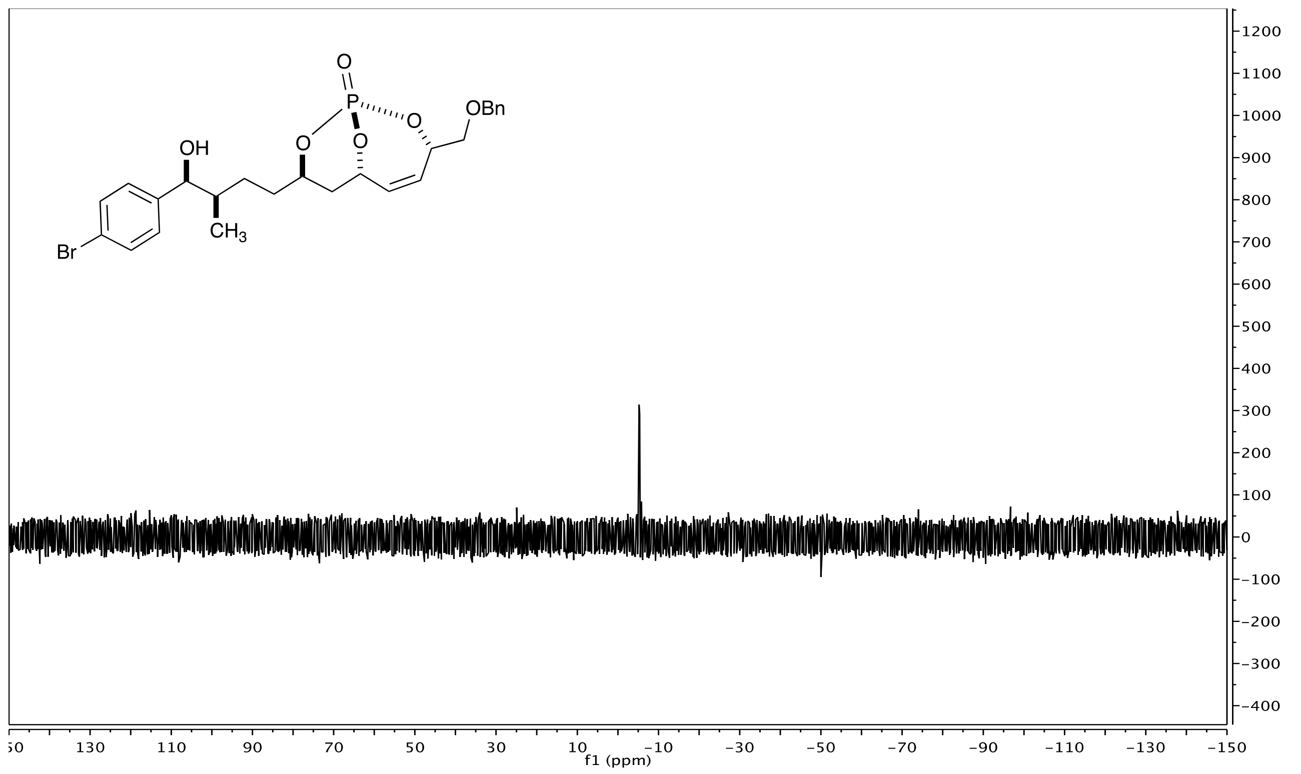

O
P
O
O
O
OBn
OH
Br
CH3
f1 (ppm)

(1*S*,2*R*,5*S*,7*R*,10*S*,*Z*)-11-(benzyloxy)-1-(4-bromophenyl)-2-methylundec-3-ene-1,5,7,10-tetraol (10)

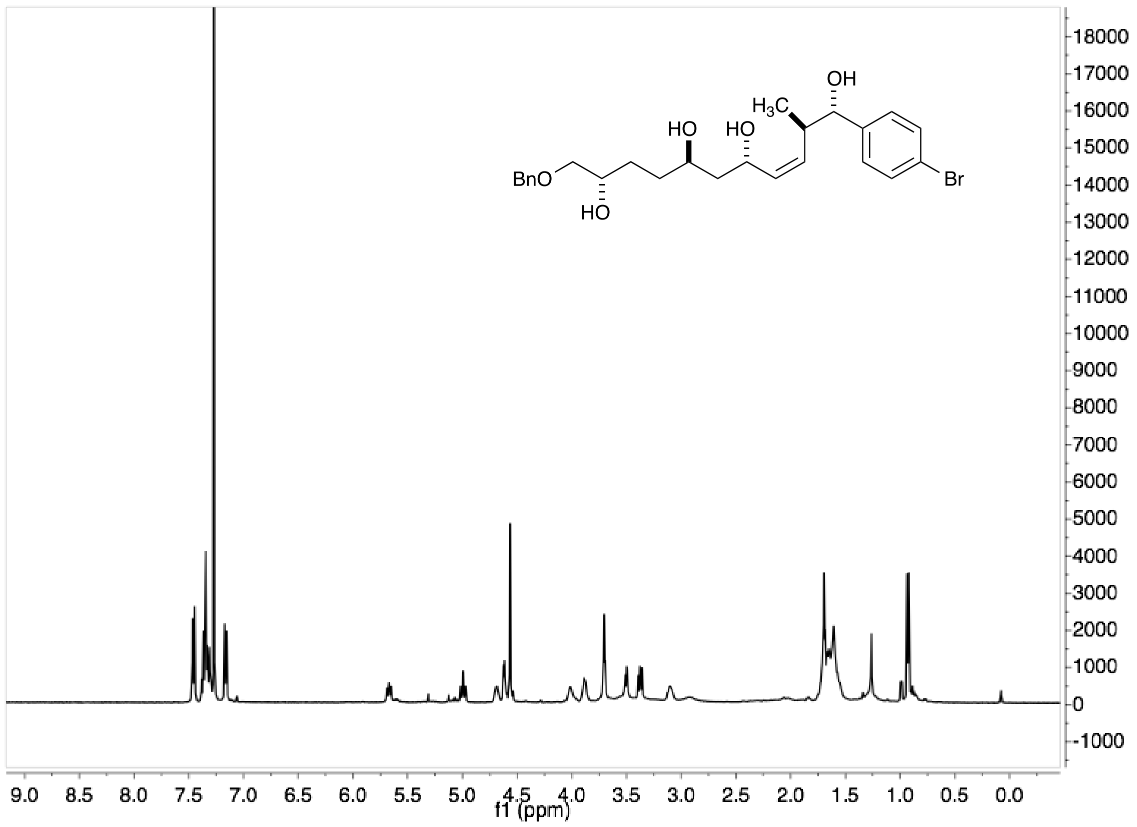

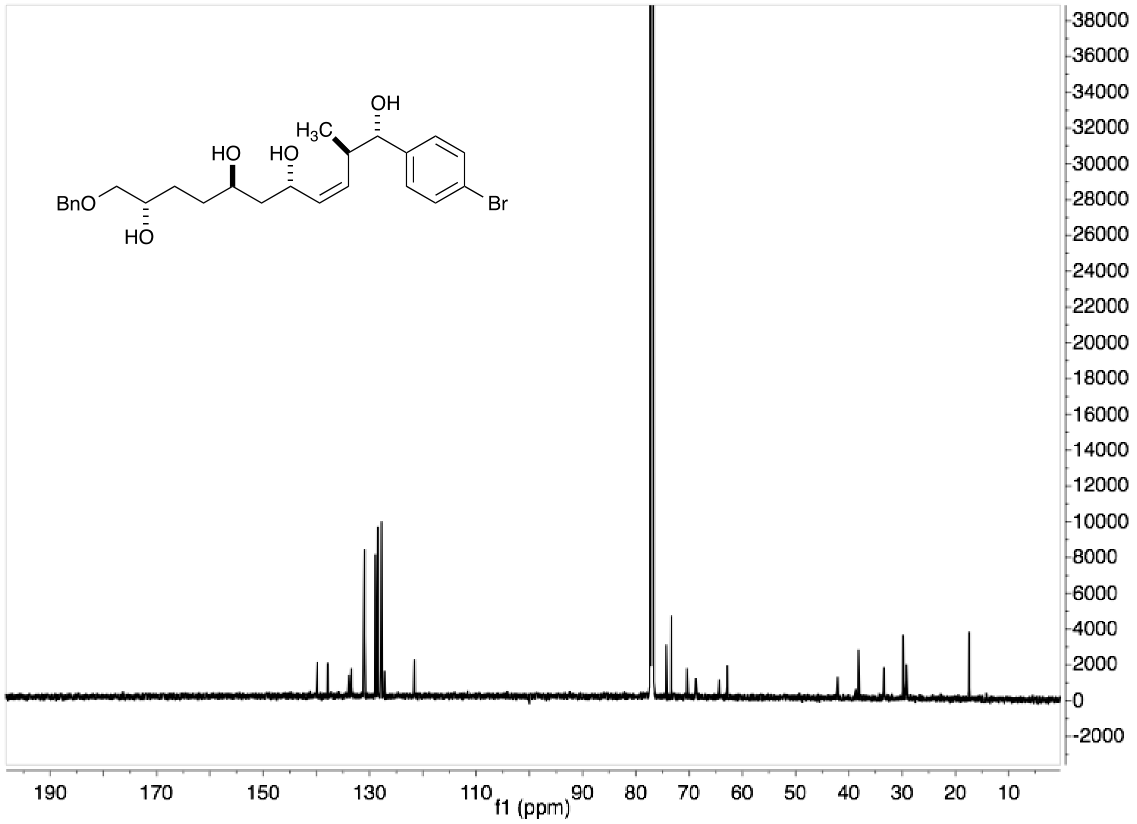

(1*S*,2*R*,5*R*,7*S*,10*S*,*Z*)-11-(benzyloxy)-1-(4-bromophenyl)-2-methylundec-8-ene-1,5,7,10-tetraol (11)

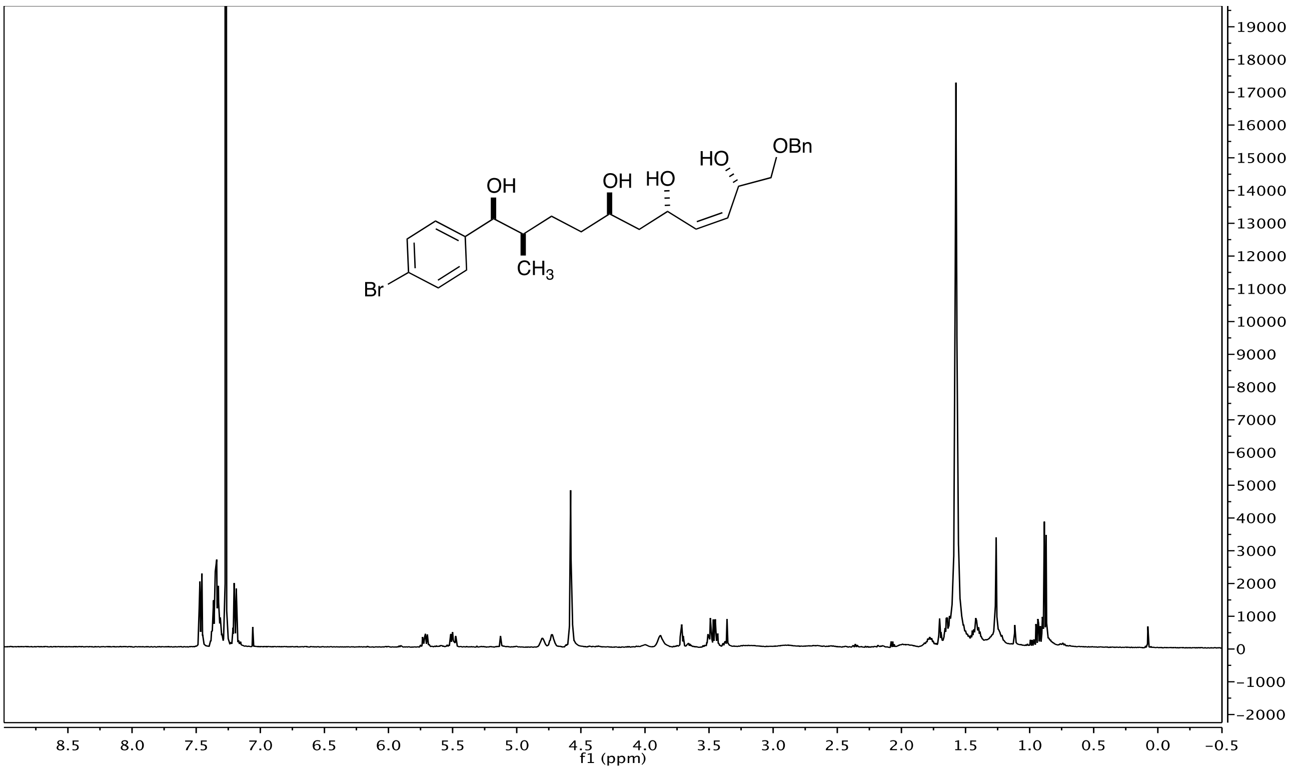

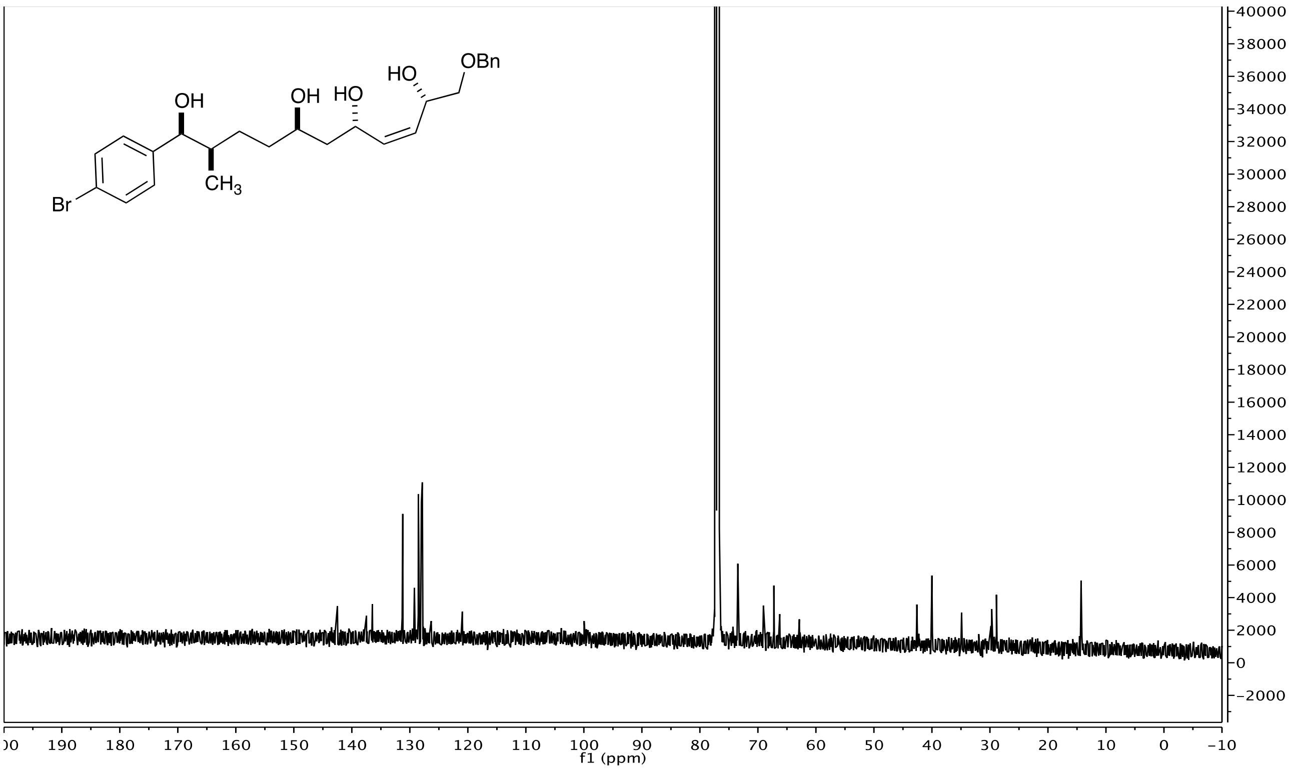

(1*S*,2*R*,3*Z*,5*S*,7*S*,8*E*,10*S*)-11-(benzyloxy)-1-(4-bromophenyl)-2-methylundeca-3,8-diene-1,5,7,10-tetraol (12)

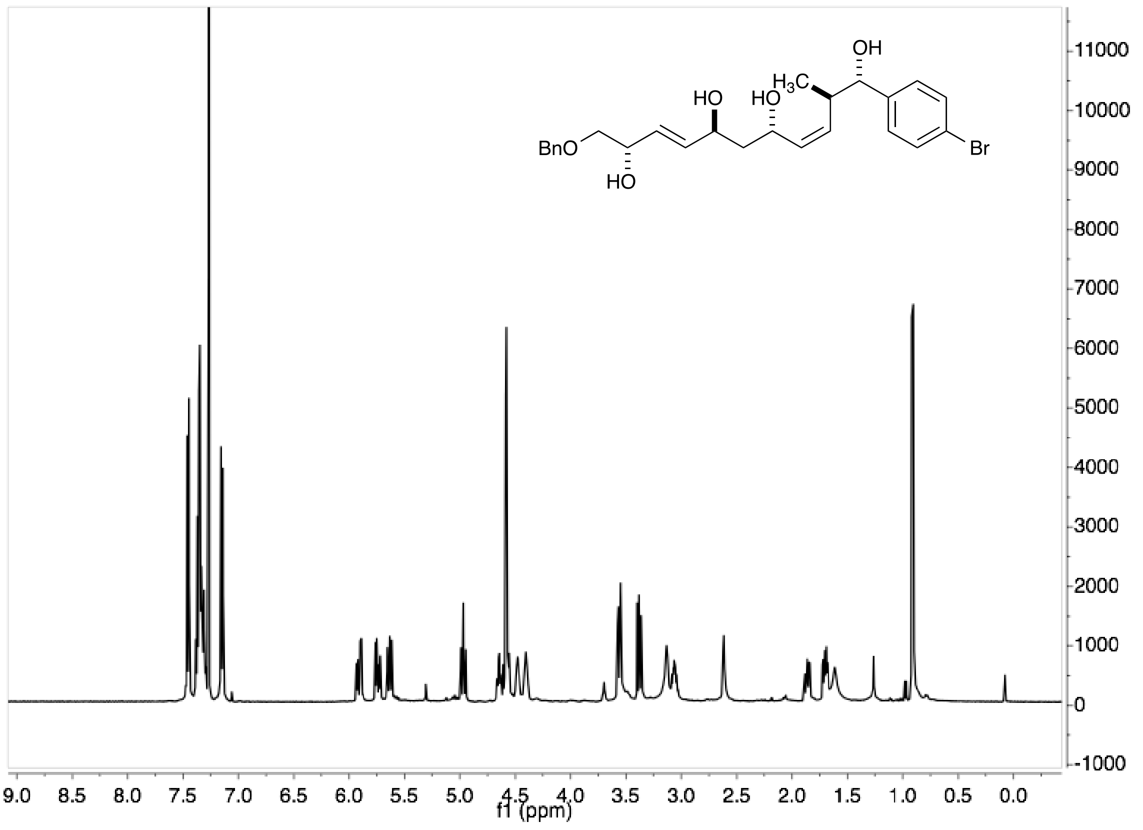

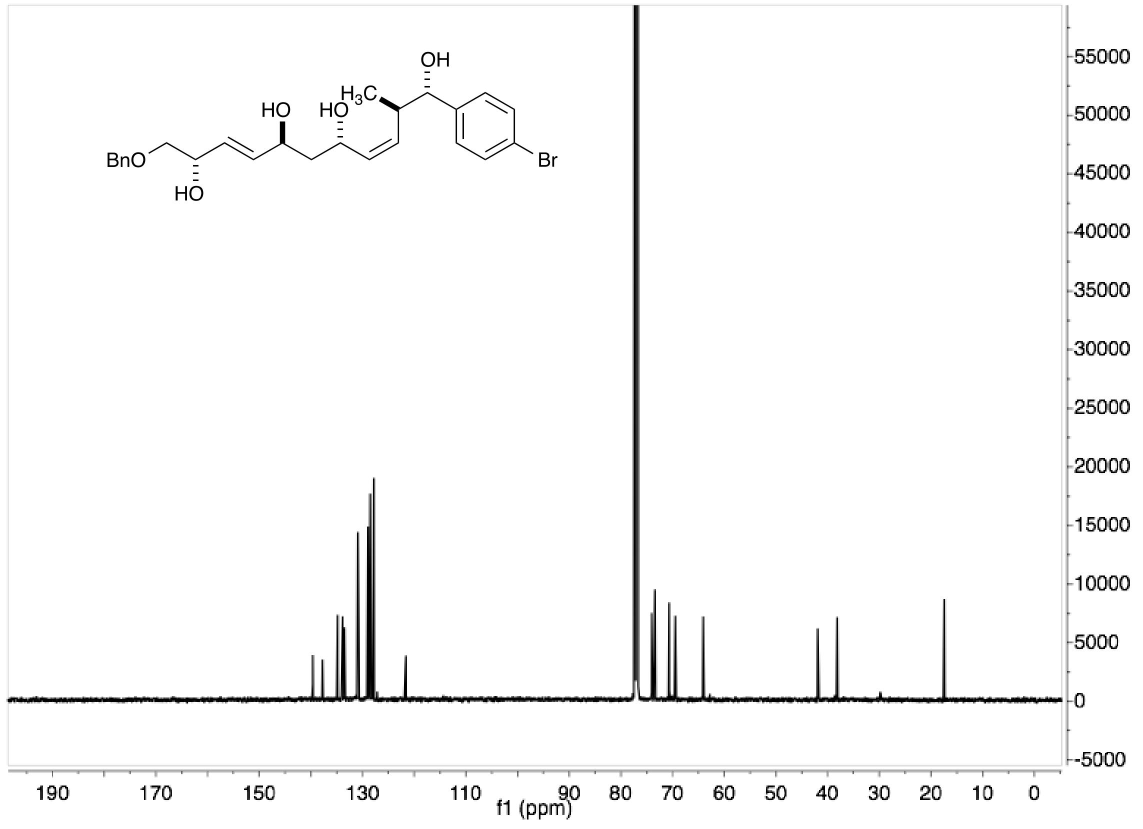

(1*S*,2*R*,3*E*,5*S*,7*S*,8*Z*,10*S*)-11-(benzyloxy)-1-(4-bromophenyl)-2-methylundeca-3,8-diene-1,5,7,10-tetraol (13)

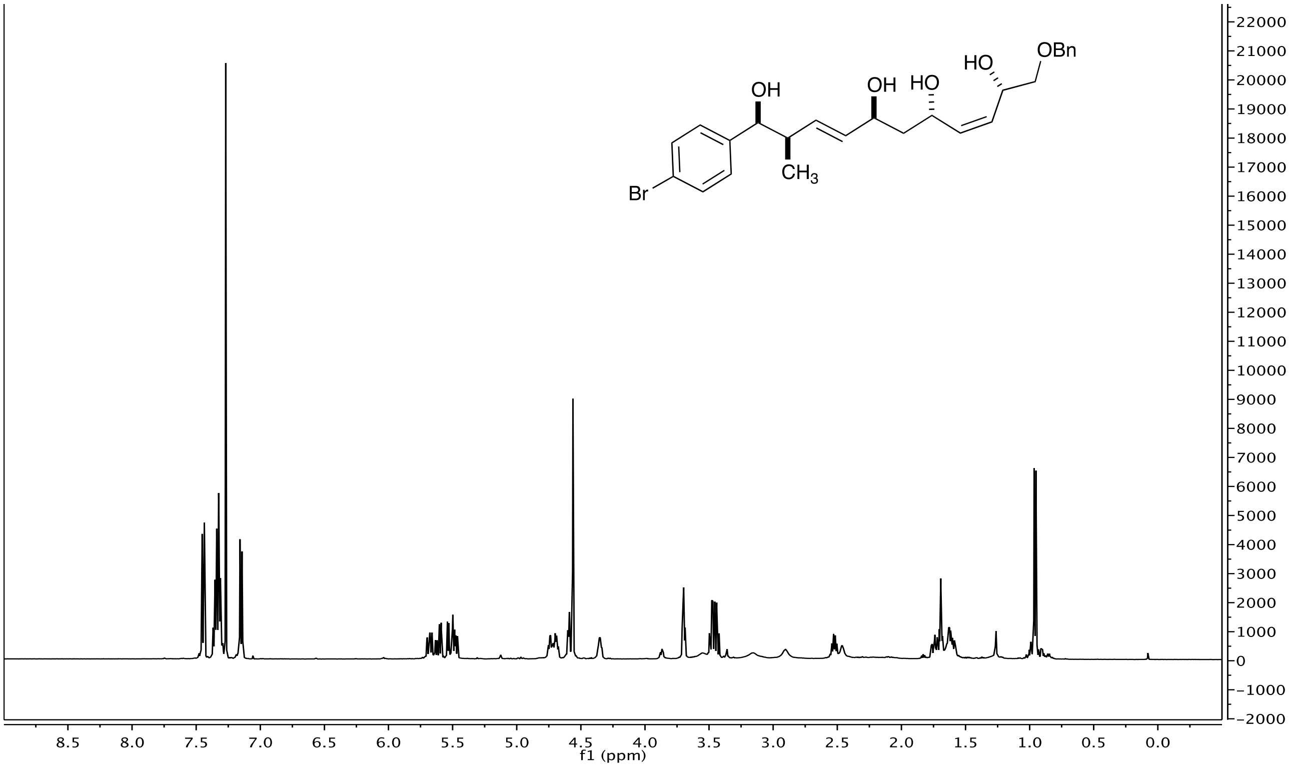

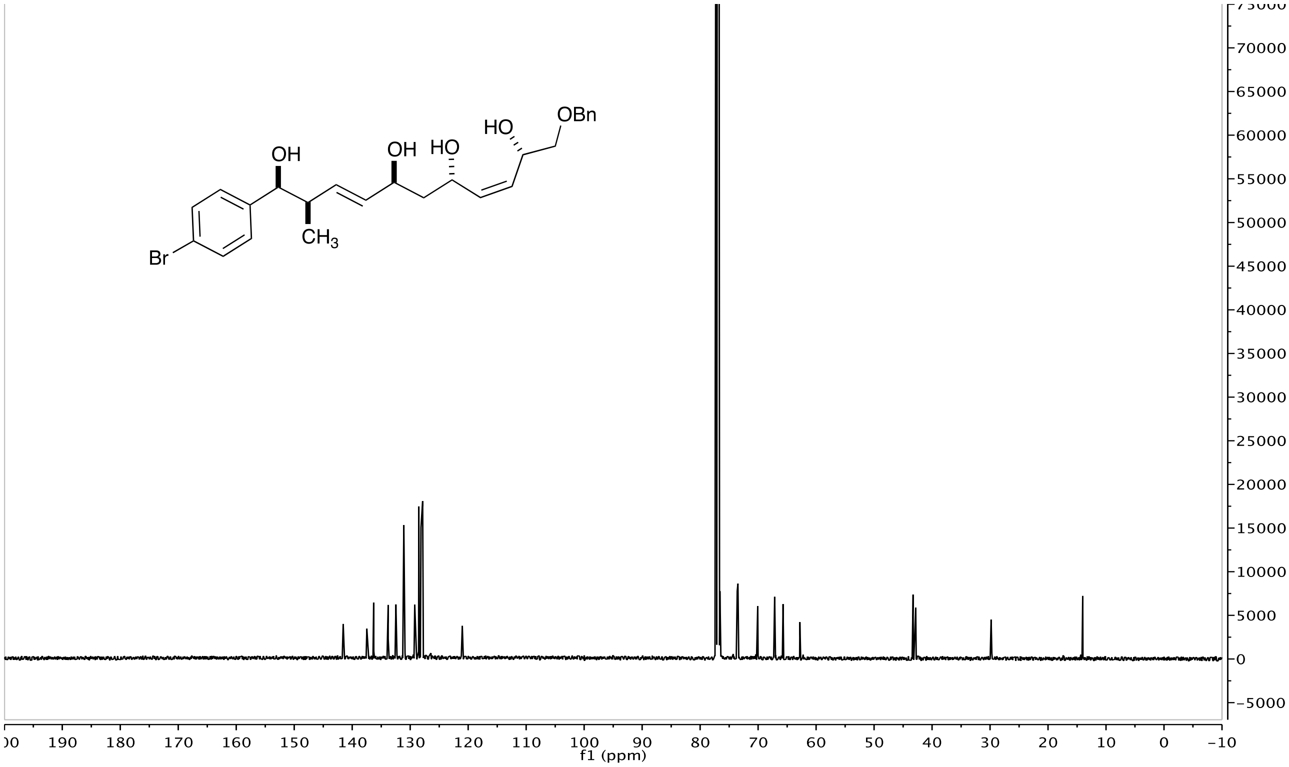

(1*S*,2*R*,5*R*,7*R*,10*S*)-11-(benzyloxy)-1-(4-bromophenyl)-2-methylundecane-1,5,7,10-tetraol (14)

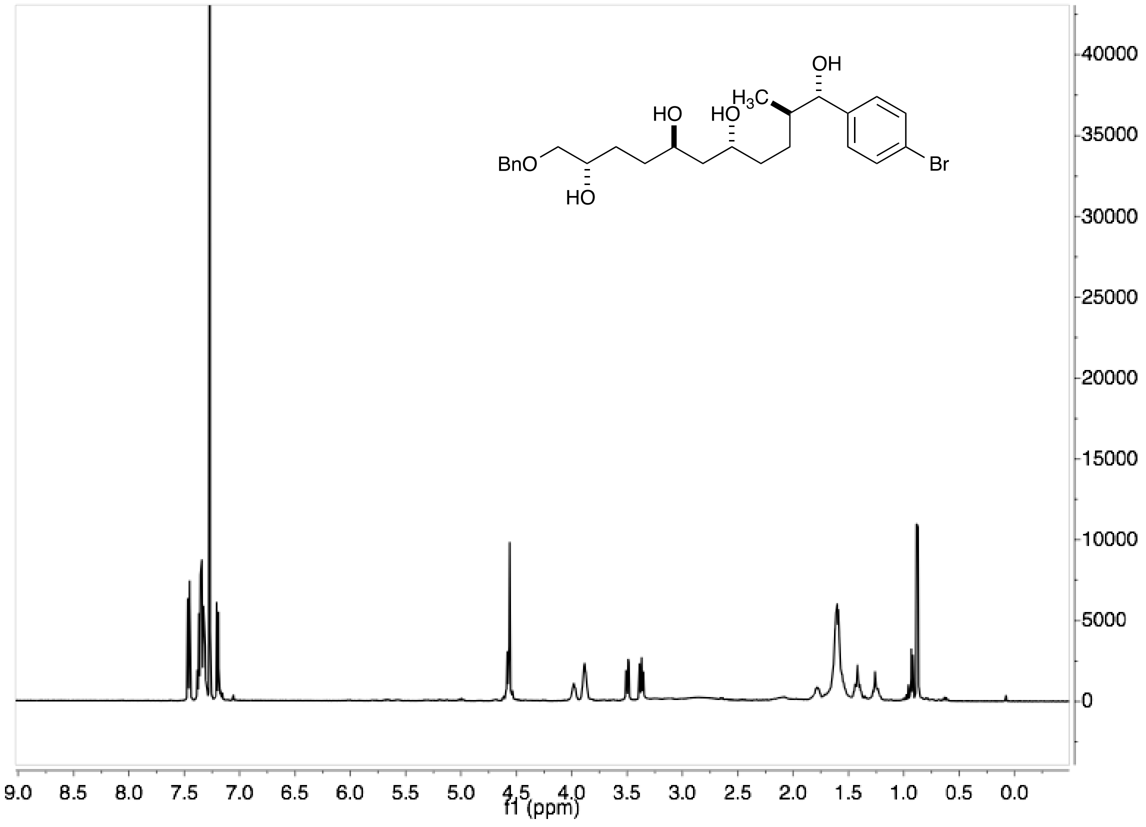

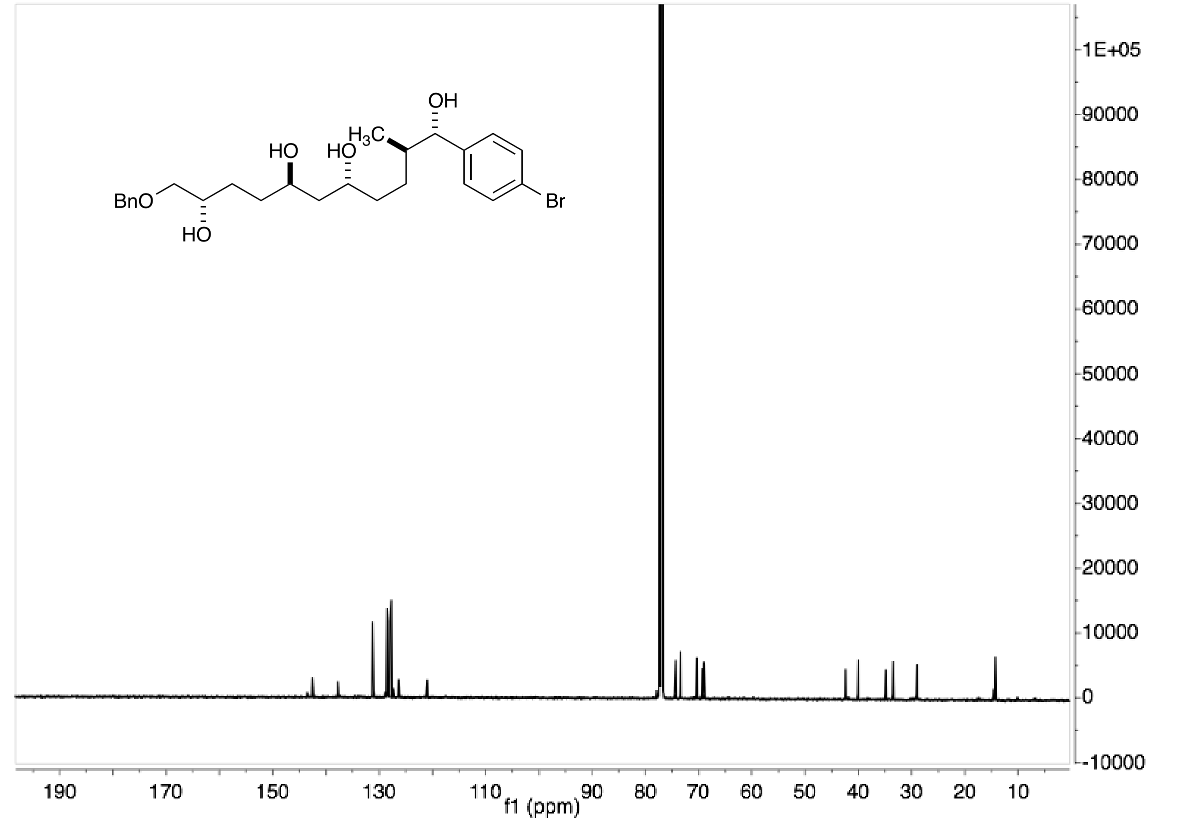

(1*S*,3*S*,7*S*,9*R*,*Z*)-9-((*S*)-4-(benzyloxy)-3-hydroxybutyl)-3-((benzyloxy)methyl)-2,10,11-trioxa-1-phosphabicyclo[5.3.1]undec-5-ene 1-oxide (15)

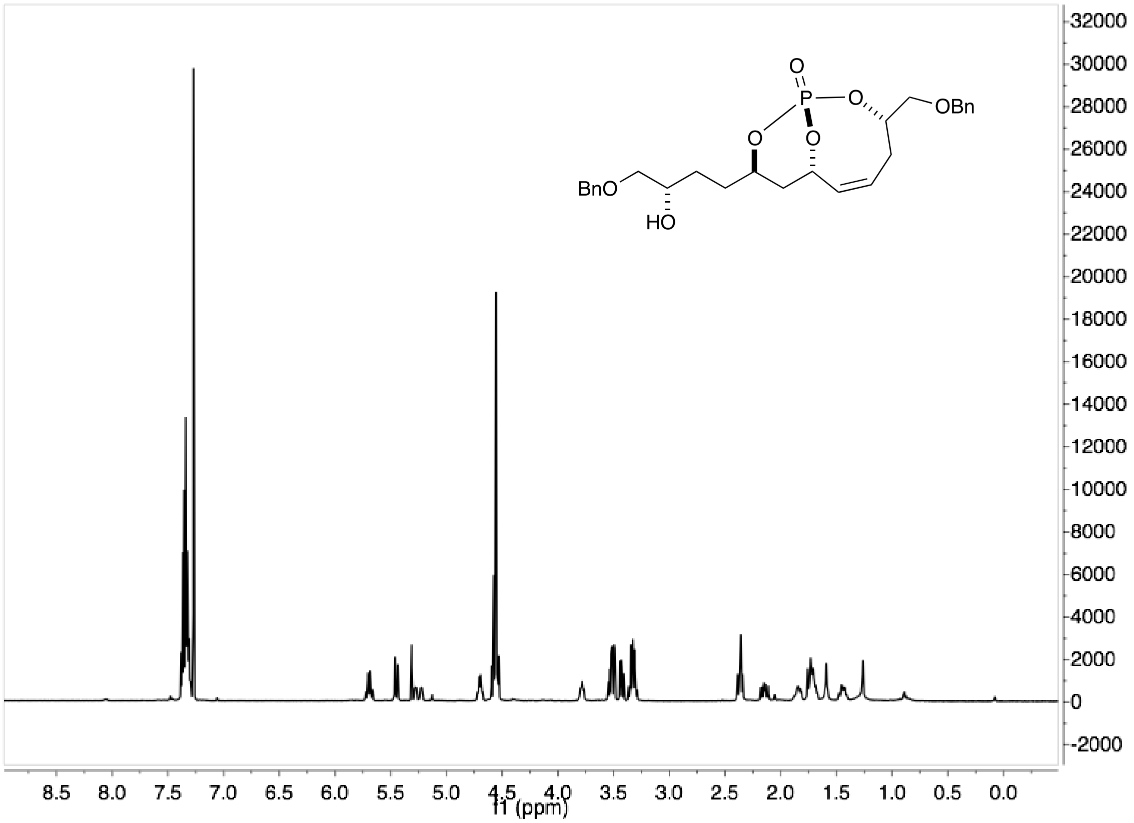

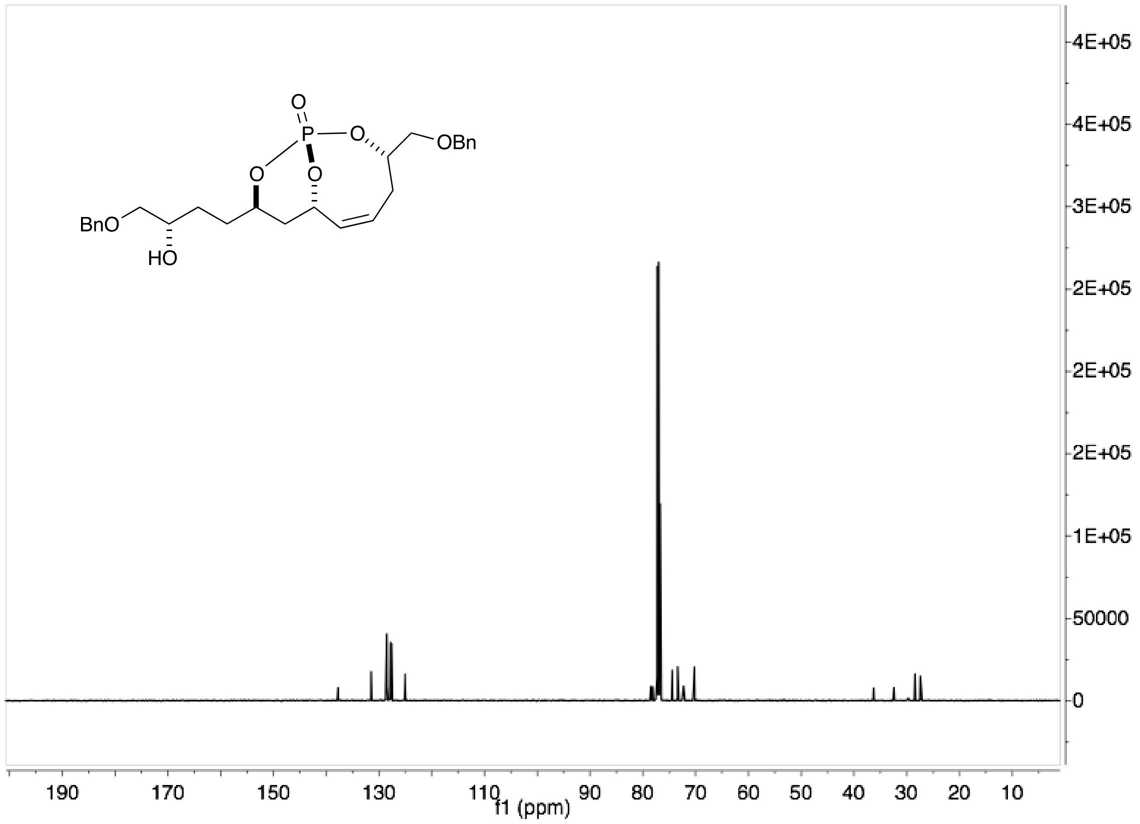

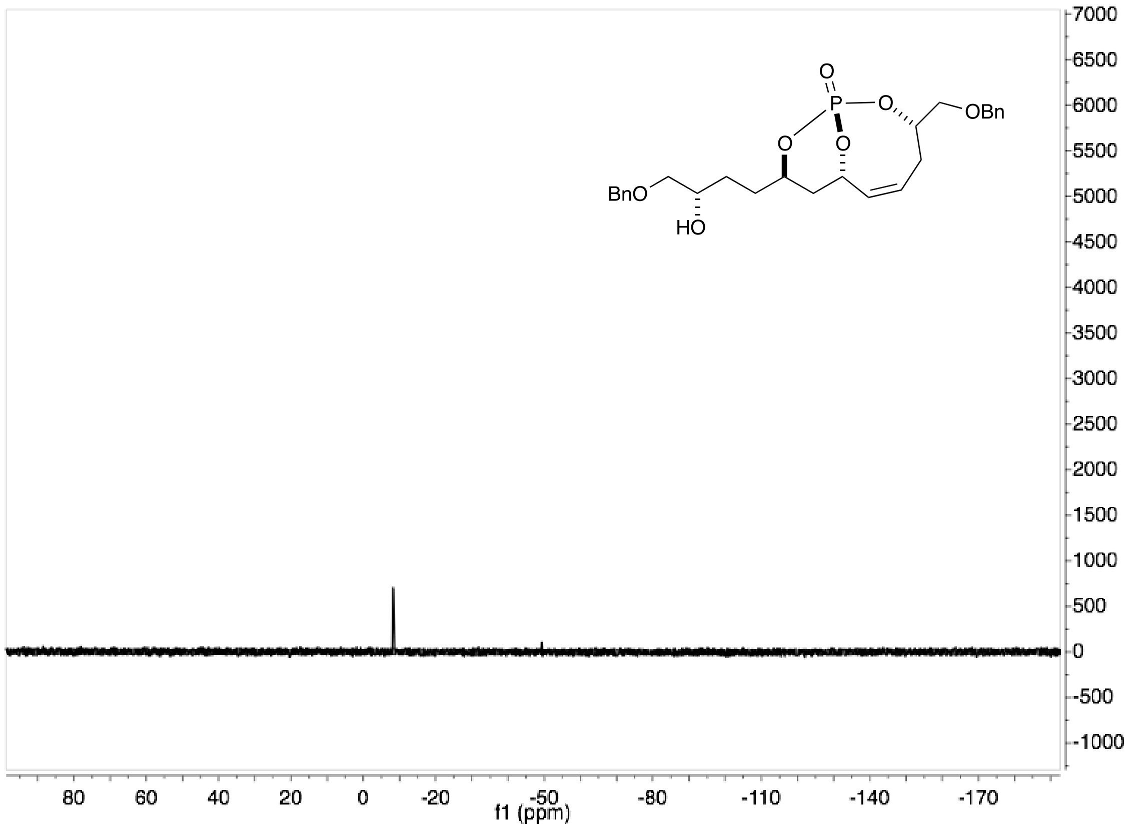

(1*S*,3*S*,6*S*,8*R*)-8-((*S*)-5-(benzyloxy)-4-hydroxypentyl)-3-((benzyloxy)methyl)-2,9,10-trioxa-1-phosphabicyclo[4.3.1]dec-4-ene 1-oxide (16)

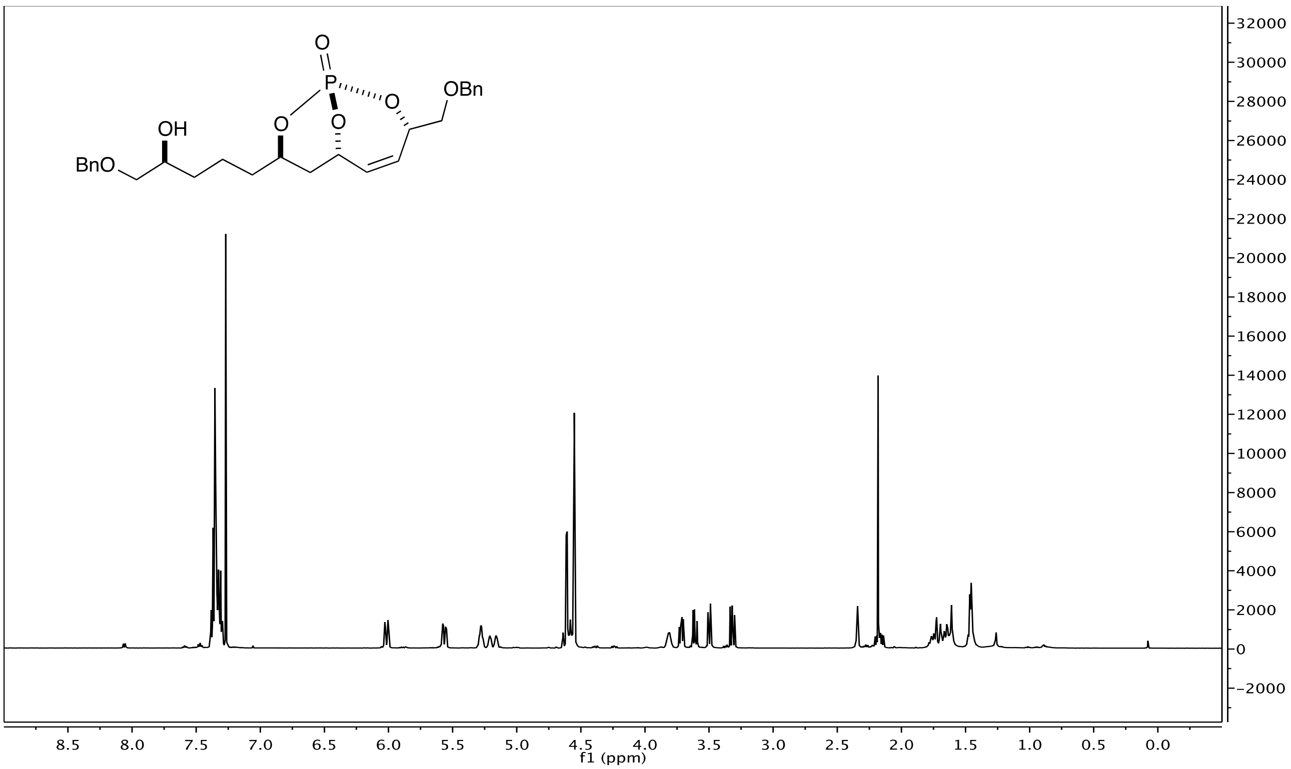

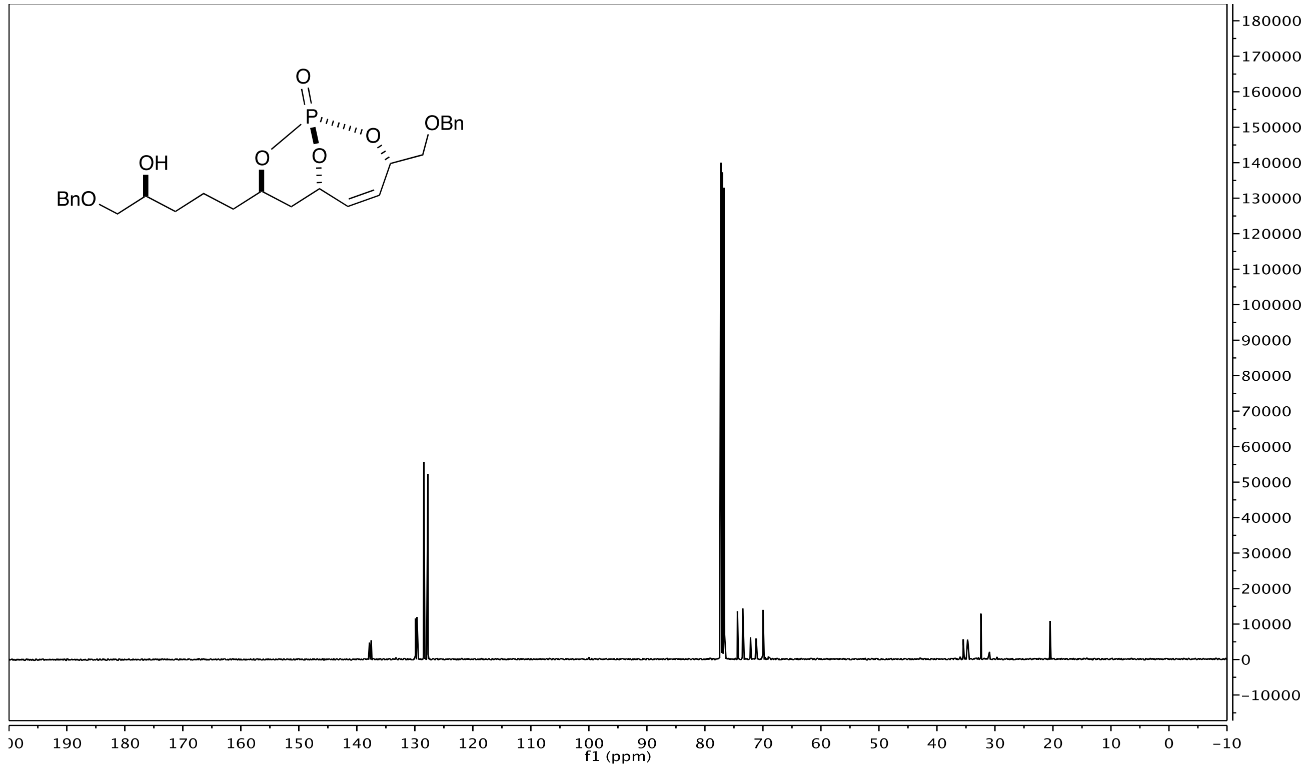

O
P
O
OBn
OH
O
O
BnO
f1 (ppm)

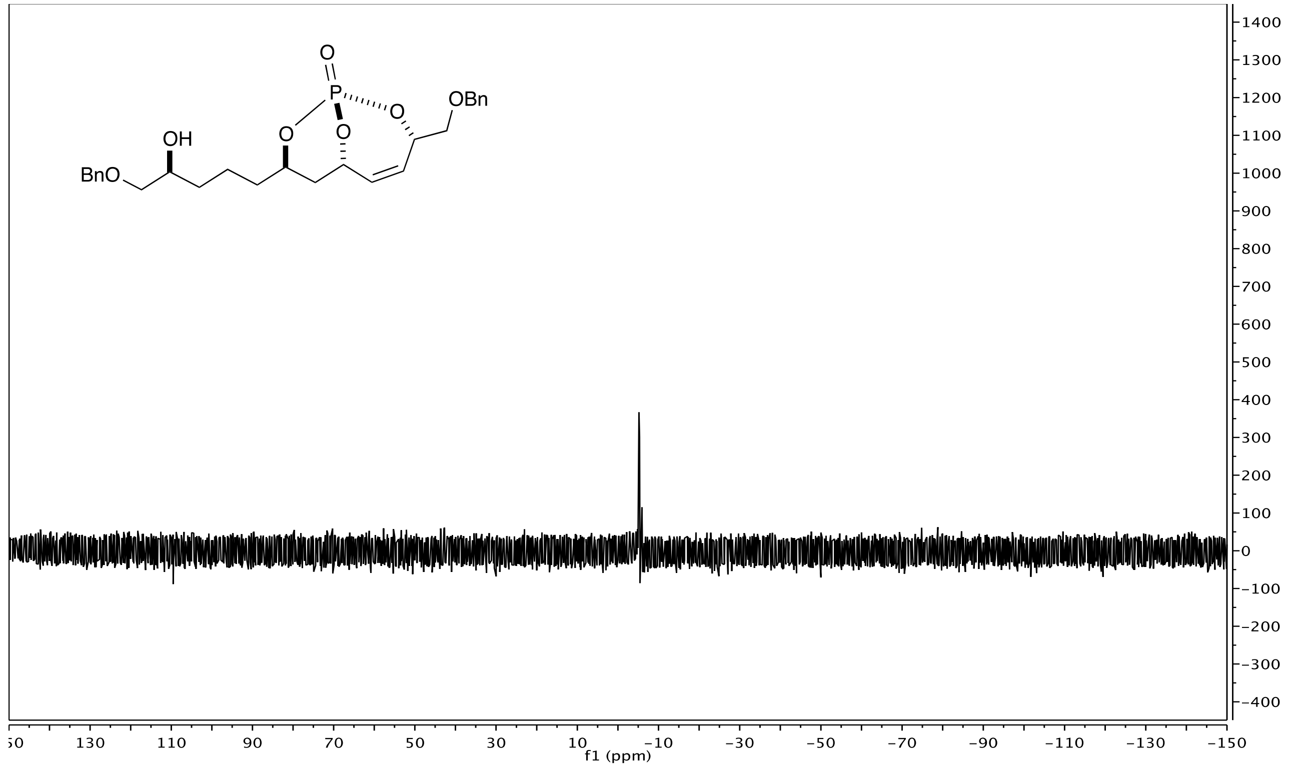

O
P
O
OBn
OH
O
O
BnO
f1 (ppm)

(2*S*,5*R*,7*S*,11*S*,*Z*)-1,12-bis(benzyloxy)dodec-8-ene-2,5,7,11-tetraol (17)

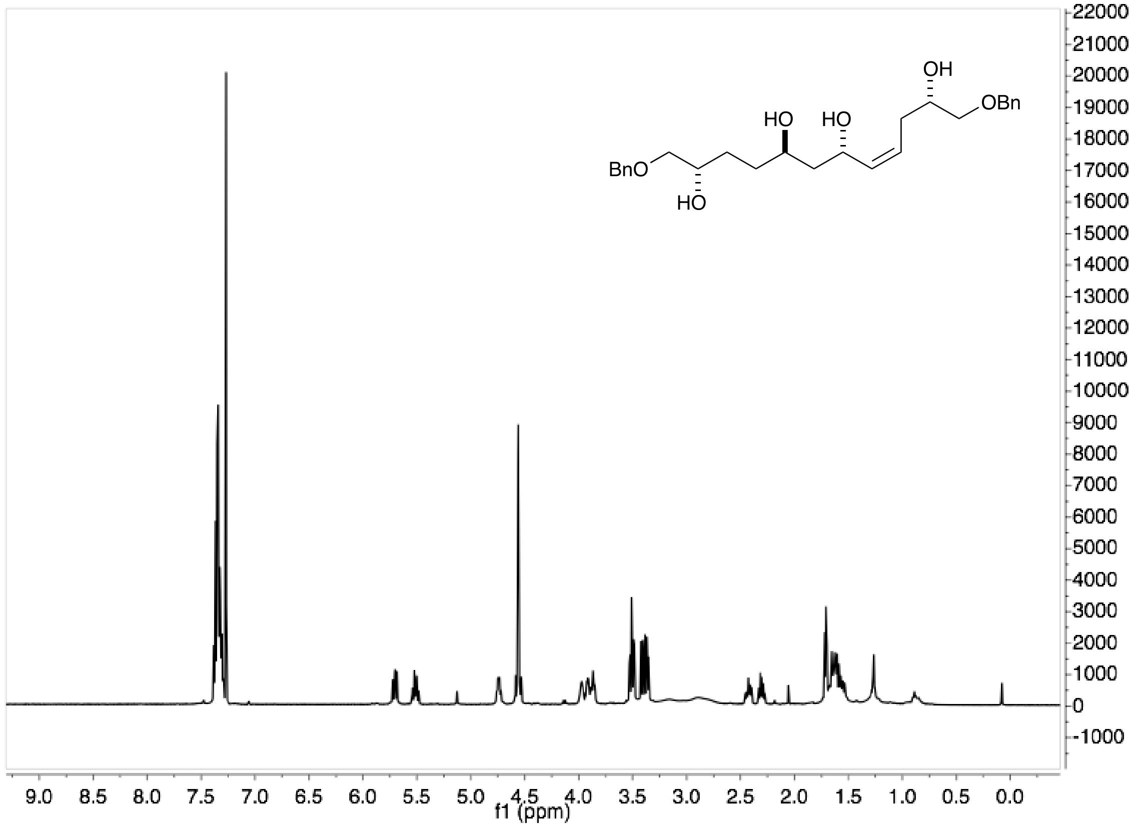

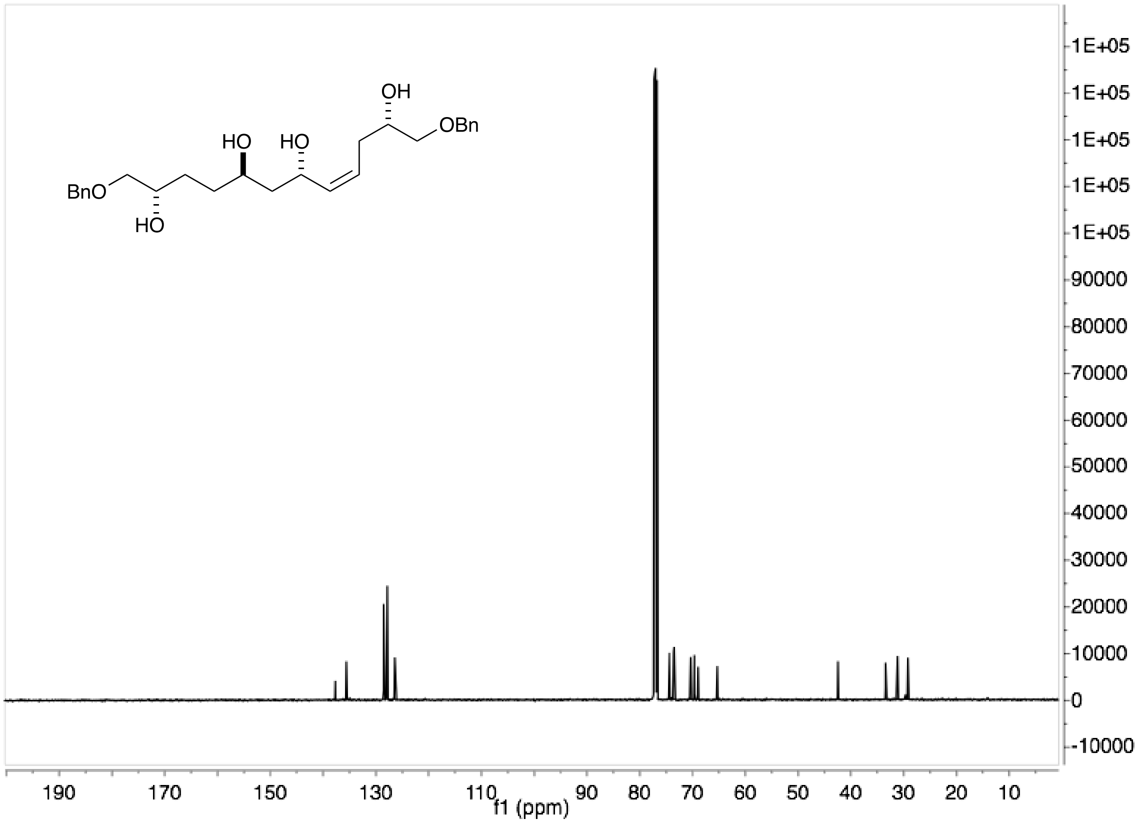

(2*S*,5*S*,7*R*,11*S*,*Z*)-1,12-bis(benzyloxy)dodec-3-ene-2,5,7,11-tetraol (18)

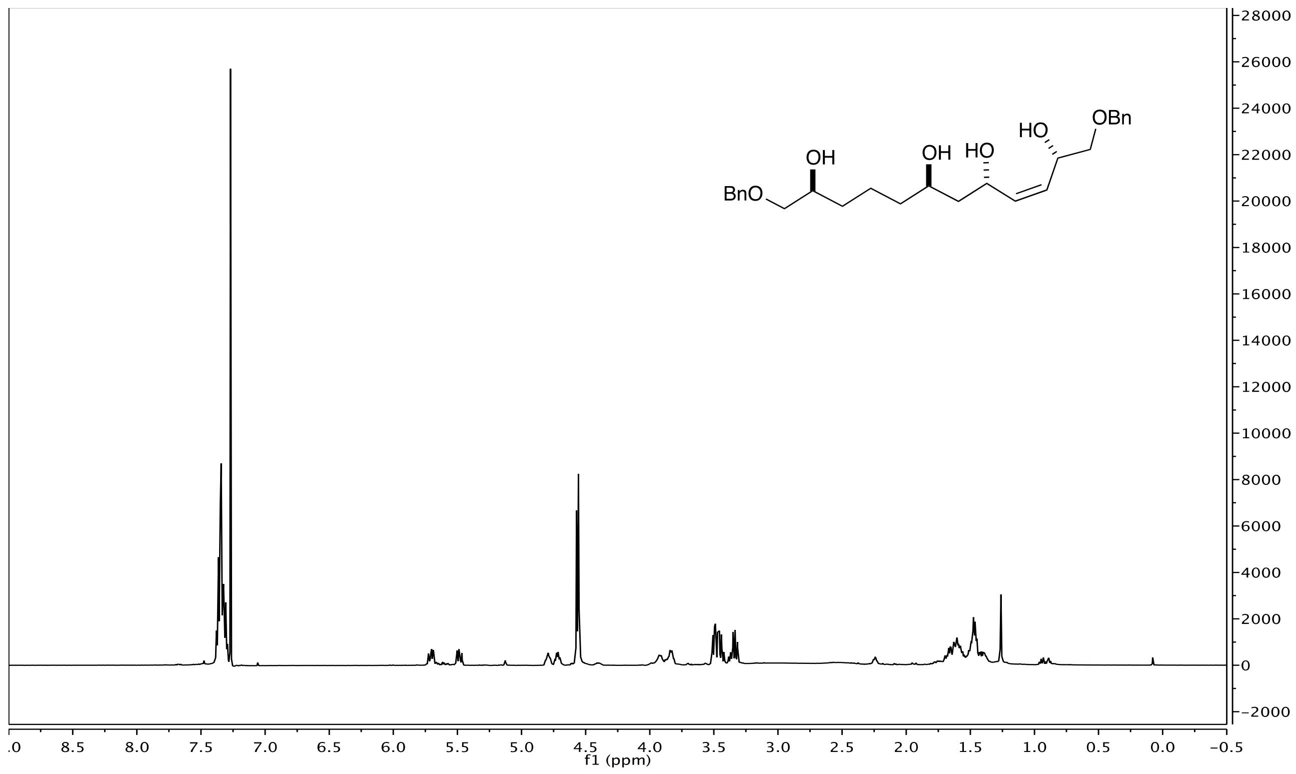

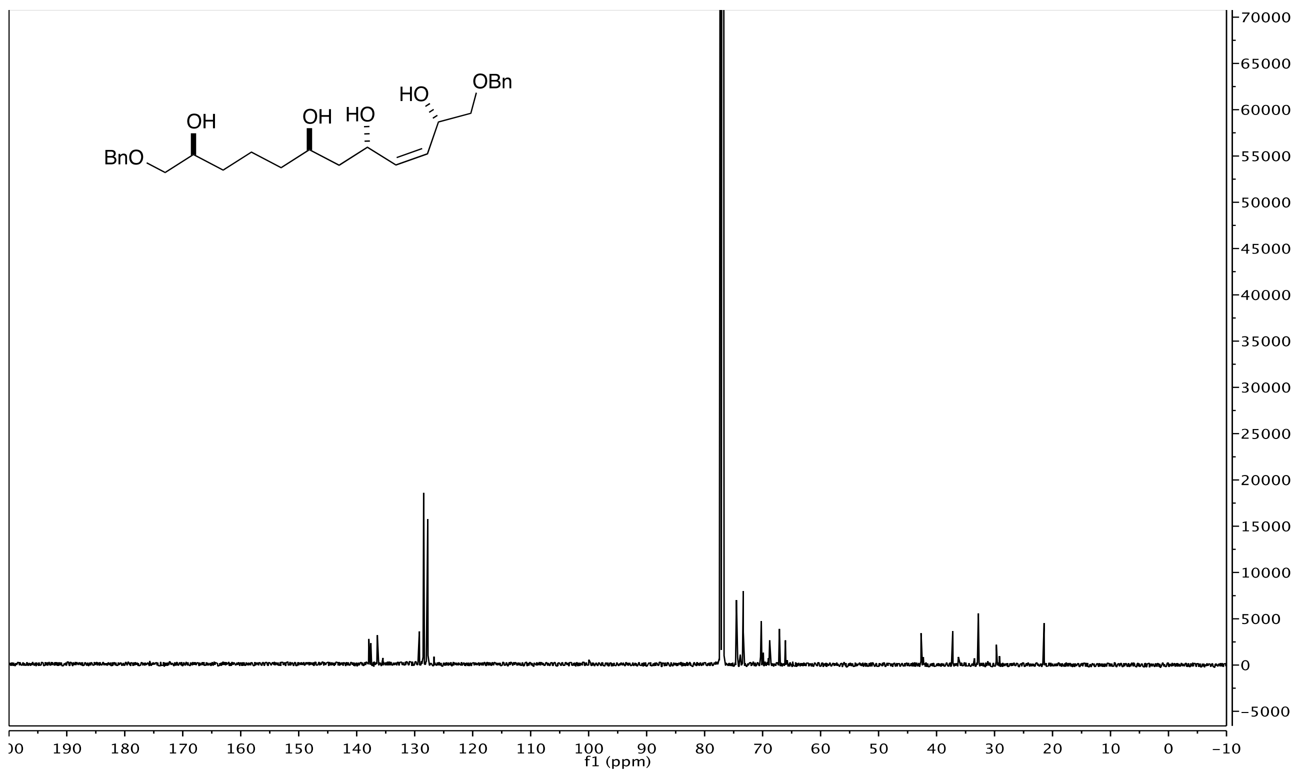

(2*S*,3*E*,5*S*,7*S*,8*Z*,11*S*)-1,12-bis(benzyloxy)dodeca-3,8-diene-2,5,7,11-tetraol (19)

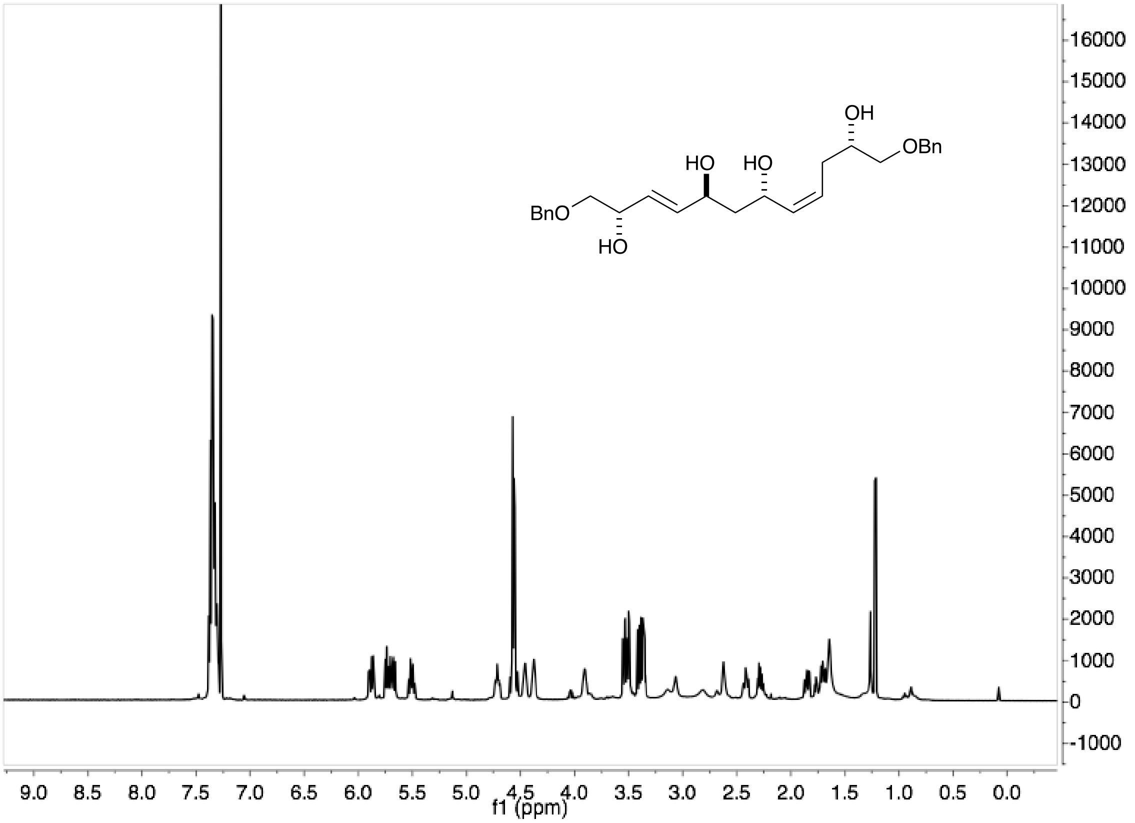

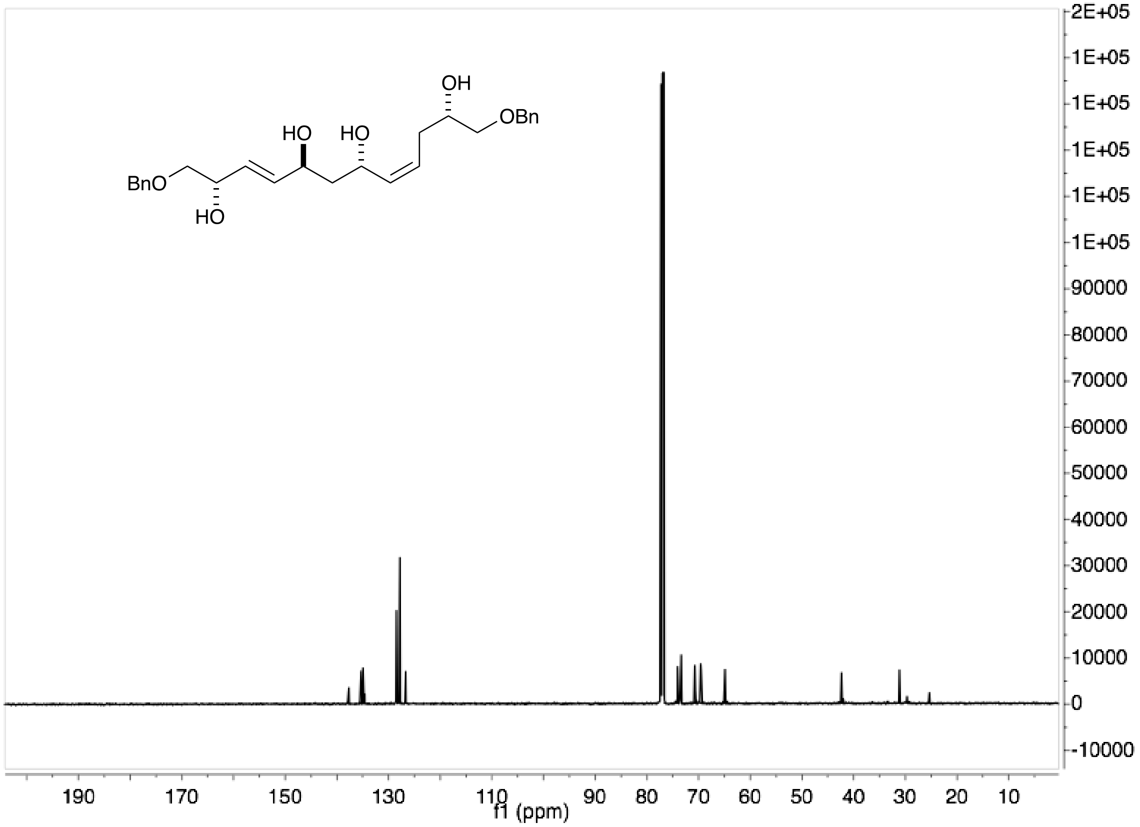

(2*S*,3*Z*,5*S*,7*S*,8*E*,11*S*)-1,12-bis(benzyloxy)dodeca-3,8-diene-2,5,7,11-tetraol (20)

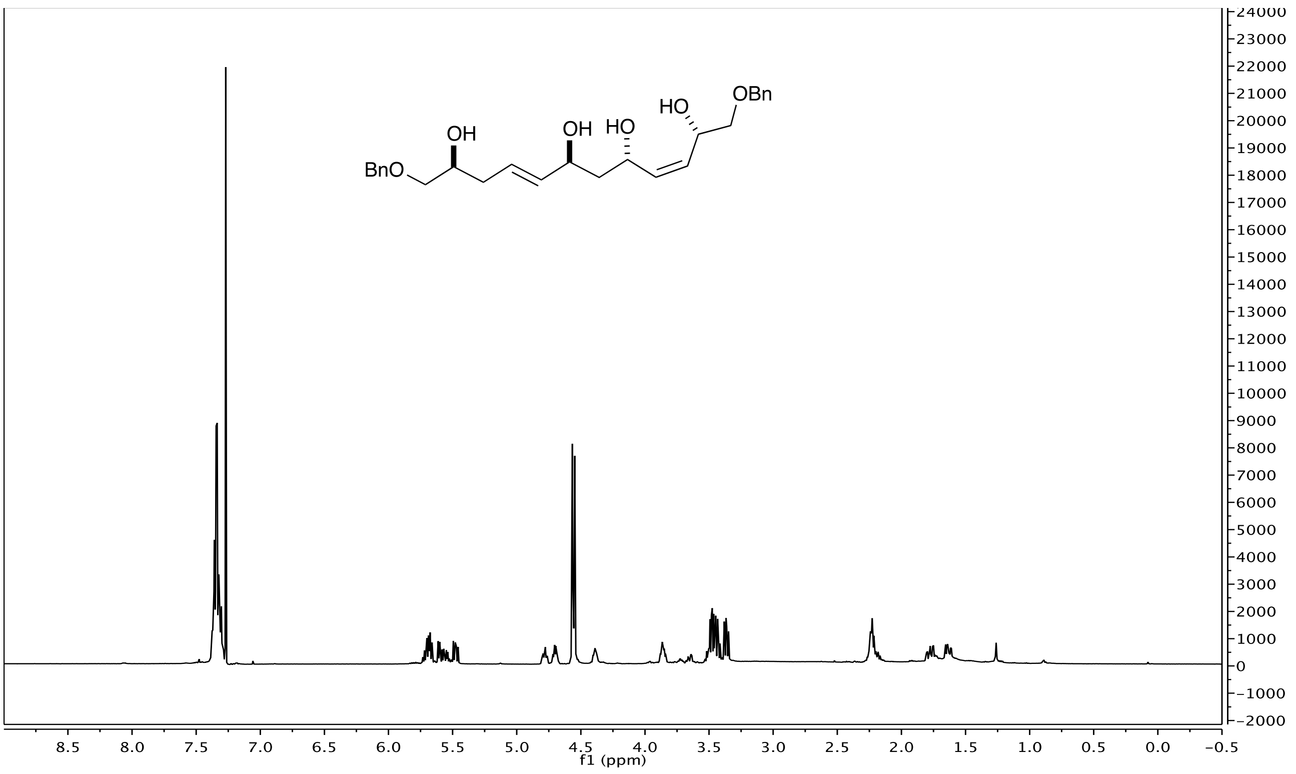

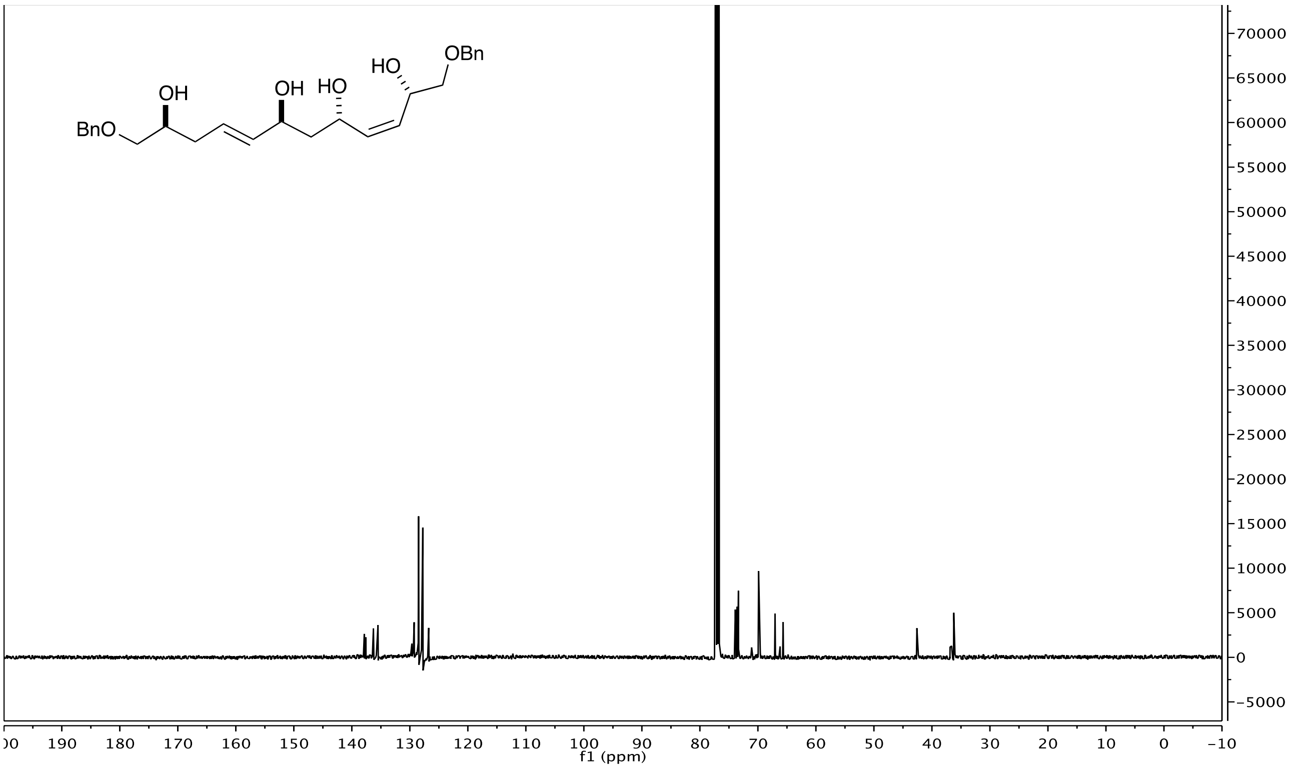

(2*S*,5*R*,7*R*,11*S*)-1,12-bis(benzyloxy)dodecane-2,5,7,11-tetraol (21)

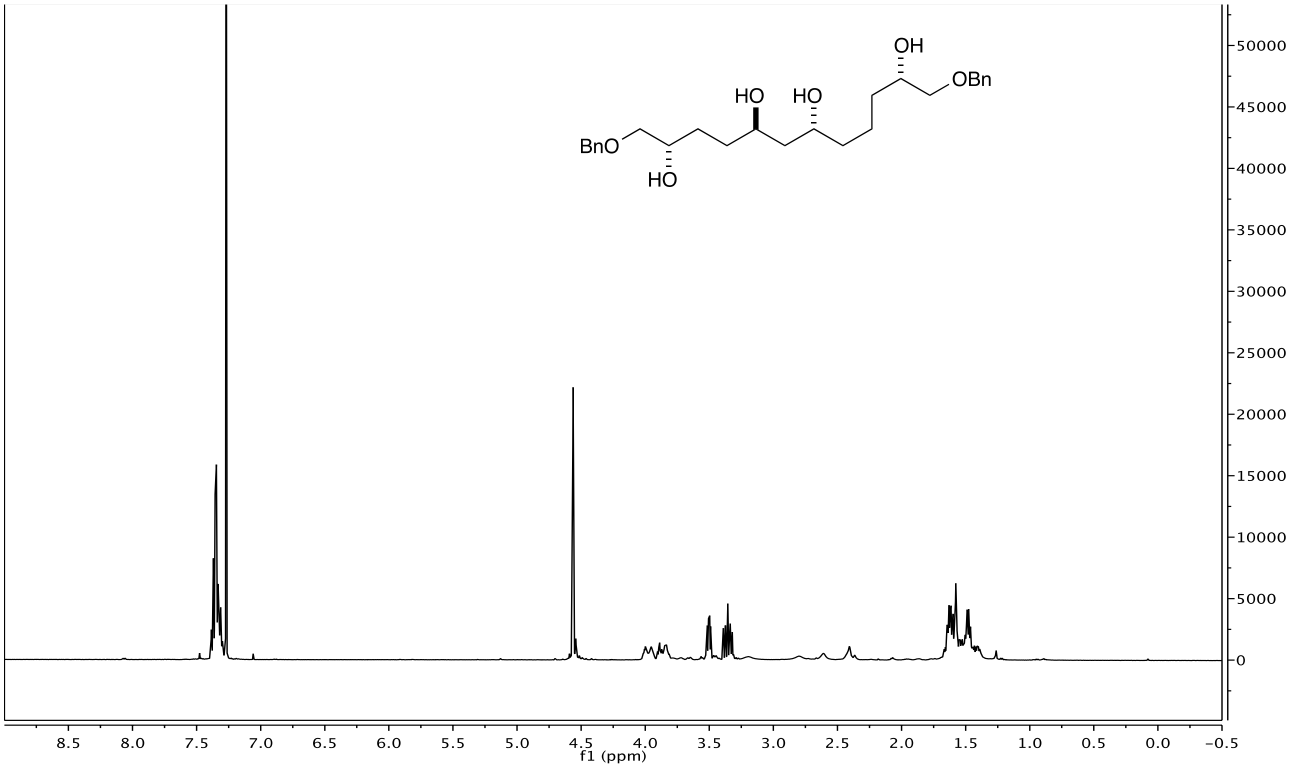

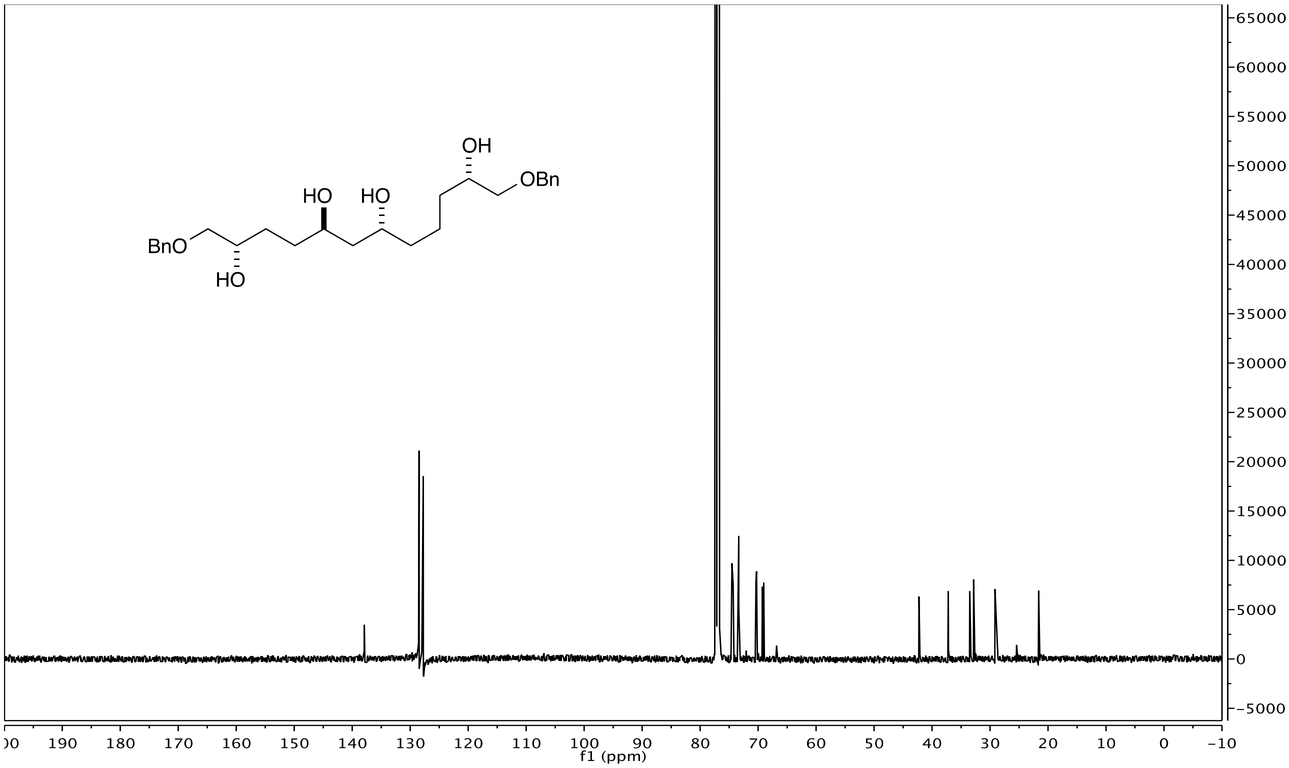

Supplement: File 1 — Experimental section. [file Beilstein_J_Org_Chem-10-2332-s001.pdf]
